# Transcriptome analysis of heterogeneity in mouse model of metastatic breast cancer

**DOI:** 10.1186/s13058-021-01468-x

**Published:** 2021-09-27

**Authors:** Anastasia A. Ionkina, Gabriela Balderrama-Gutierrez, Krystian J. Ibanez, Steve Huy D. Phan, Angelique N. Cortez, Ali Mortazavi, Jennifer A. Prescher

**Affiliations:** 1 Department of Molecular Biology and Biochemistry, University of California Irvine, Irvine, CA 92697 USA; 2 Department of Developmental and Cell Biology, University of California Irvine, Irvine, CA 92697 USA; 3 Center for Complex Biological Systems, University of California Irvine, Irvine, CA 92697 USA; 4 Department of Chemistry, University of California Irvine, Irvine, CA 92697 USA; 5 Department of Pharmaceutical Sciences, University of California Irvine, Irvine, CA 92697 USA

**Keywords:** Breast cancer, Tumor heterogeneity, Metastasis, Single-cell RNA sequencing, Organ tropism, MMTV-PyMT model

## Abstract

**Background:**

Cancer metastasis is a complex process involving the spread of malignant cells from a primary tumor to distal organs. Understanding this cascade at a mechanistic level could provide critical new insights into the disease and potentially reveal new avenues for treatment. Transcriptome profiling of spontaneous cancer models is an attractive method to examine the dynamic changes accompanying tumor cell spread. However, such studies are complicated by the underlying heterogeneity of the cell types involved. The purpose of this study was to examine the transcriptomes of metastatic breast cancer cells using the well-established MMTV-PyMT mouse model.

**Methods:**

Organ-derived metastatic cell lines were harvested from 10 female MMTV-PyMT mice. Cancer cells were isolated and sorted based on the expression of CD44^low^/EpCAM^high^ or CD44^high^/EpCAM^high^ surface markers. RNA from each cell line was extracted and sequenced using the NextSeq 500 Illumina platform. Tissue-specific genes were compared across the different metastatic and primary tumor samples. Reads were mapped to the mouse genome using STAR, and gene expression was quantified using RSEM. Single-cell RNA-seq (scRNA-seq) was performed on select samples using the ddSeq platform by BioRad and analyzed using Seurat v3.2.3. Monocle2 was used to infer pseudo-time progression.

**Results:**

Comparison of RNA sequencing data across all cell populations produced distinct gene clusters. Differential gene expression patterns related to CD44 expression, organ tropism, and immunomodulatory signatures were observed. scRNA-seq identified expression profiles based on tissue-dependent niches and clonal heterogeneity. These cohorts of data were narrowed down to identify subsets of genes with high expression and known metastatic propensity. Dot plot analyses further revealed clusters expressing cancer stem cell and cancer dormancy markers. Changes in relevant genes were investigated across pseudo-time and tissue origin using Monocle2. These data revealed transcriptomes that may contribute to sub-clonal evolution and treatment evasion during cancer progression.

**Conclusions:**

We performed a comprehensive transcriptome analysis of tumor heterogeneity and organ tropism during breast cancer metastasis. These data add to our understanding of metastatic progression and highlight targets for breast cancer treatment. These markers could also be used to image the impact of tumor heterogeneity on metastases.

**Supplementary Information:**

The online version contains supplementary material available at 10.1186/s13058-021-01468-x.

## Background

Despite recent advances in treatment and diagnosis, metastatic breast cancer remains a leading cause of death for women worldwide [1]. Cancer metastasis is a complex process involving the spread of malignant cells from a primary tumor to distal organs [2, 3]. Premalignant cells undergo dynamic cellular changes (i.e., epithelial to mesenchymal transition, EMT) to escape the primary tumor [3, 4]. These same cells undergo the reverse process (i.e., mesenchymal to epithelial transition, MET) to colonize metastatic sites [5]. Expression and fluctuations of cell surface markers (e.g., CD44) have long been associated with metastatic progression in breast cancer [4]. However, exactly which cells within a given primary tumor ultimately metastasize—and their final destinations—remains unclear [4–6].

Transcriptome profiling of the dynamic cellular changes during tumorigenesis has the potential to improve our understanding of metastatic disease. Such analyses can reveal biomarkers associated with malignant progression. In one example, bulk RNA-sequencing (RNA-seq) revealed novel molecular pathways and differentially expressed genes (DEGs) associated with distinct stages of breast cancer progression [7]. However, traditional profiling studies are complicated by the underlying heterogeneity of cancer progression. The contribution of distinct cell populations cannot be discerned using bulk RNA-seq alone [8]. Single-cell RNA-seq (scRNA-seq) technology captures the complexity of cellular heterogeneity by mapping transcripts to individual cells [9]. This increase in cellular resolution facilitates the identification of additional molecular pathways and cell specific biomarkers [10, 11].

Examining breast cancer remains challenging owing to a lack of models that capture cellular heterogeneity [12]. The surrounding microenvironment, cancer stem cells (CSCs), and tumor dormancy all contribute to disease progression beyond isolated changes to the malignant cells themselves. These features are difficult to replicate outside of living organisms. Suitable models must take into account the different tissue microenvironments that support cancer niches and resident cancer stem cells during metastatic progression [13–16]. The MMTV-PyMT mouse model, in particular, is a well-established platform to study human breast cancer [17, 18]. However, the variability in different metastatic niches and the contribution of different cancer cell types to disease progression remain unclear. Subclones across breast tumors are frequently identified and monitored using the expression of the cell surface marker CD44 [4–6]. However, this marker is associated with both pro- and anti-tumorigenic outcomes, meaning that CD44 expression alone cannot be used to predict metastatic propensity or other cell behaviors [19–22]. Transcriptome profiling of the MMTV-PyMT cancer model could thus provide more insight into the mechanisms underlying dynamic changes in tumor progression [23].

We aimed to understand the transcriptome changes of organ-derived cancer cell isolates from MMTV-PyMT mice. Although metastatic progression from primary tumors to lung tissue is well studied in the MMTV-PyMT model, metastases to other distal organs and the significance of intratumor heterogeneity remain unclear [24]. To gain insight, we established an array of metastatic cell lines harvested from MMTV-PyMT mice. Differential expression analyses were performed and used to examine the effects of cell heterogeneity on metastases and organ tropism. Correlations were found between CD44 expression and cellular growth markers across all metastatic cells. Data from scRNA-seq analyses further revealed tissue-specific gene expression patterns that mirror clinical data. Overall, the suite of clonal isolates provided a detailed depiction of cancer progression. The cell lines also establish a platform for future studies examining heterogeneity during metastatic disease and elucidating transcriptomic changes relevant to malignancy.

## Methods

### Mammalian cell culture

Unless otherwise stated, cell lines were cultured in DMEM (Corning) supplemented with 10% (vol/vol) fetal bovine serum (FBS, Life Technologies), penicillin (100 U/mL) and streptomycin (100 µg/mL). Cells were maintained in a 5% CO_2_ water-saturated incubator at 37 °C.

### MMTV-PyMT metastatic cell lines as models of breast cancer

Mouse experiments were approved by the UC Irvine Animal Care and Use Committee. Tumor bearing organs were harvested from 10–12-week females FVB/NJ MMTV-PyMT mice (courtesy of the Kessenbrock laboratory, UCI). Samples were processed mechanically and chemically to dissociate tissues into single cell suspensions as previously published [25]. Primary single cell suspensions were enriched for cancer cells over the course of 1-month in vitro by exploiting differences in cellular nutrient requirements and growth. During this time course, primary single cell suspensions were enriched for cancer cells by culturing cell lines in 5% FBS. Cultures were selected for immortalized cancer cells in vitro by passaging the flasks 3 times a week. Differences in cellular adhesion properties between fibroblasts and epithelial cancer cells were also exploited in vitro through 3-min versus 7-min incubations with trypsin (Schor et al., 1979). The month-long process resulted in the enrichment of cancer cell lines prior to FACS sorting.

Primary cell lines were processed for FACS sorting (Institute for Immunology Flow Cytometry Core, UCI) as previously reported [26]. Cancer cells were isolated by EpCAM (BioLegend 118213) and CD44 (BioLegend 103027) cell surface expression levels [27]; isolated cancer cells expressed either CD44^low^/EpCAM^high^ or CD44^high^/EpCAM^high^ cell surface markers. Antibody labeling was performed using manufacturer protocols (BioLegend, USA). Previously sorted MMTV-PyMT MFP-eGFP cell lines (VO) (courtesy of the Kessenbrock laboratory and Lawson laboratory, UCI) were used as positive controls. Fibroblast cell lines (3T3 and MMTV-PyMT-derived fibroblasts, isolated during culturing process above) were used as negative controls during sorting.

### Primary cell line metastatic propensity validation in vivo

MFP-derived cells (100,000 cells/injection) were injected bilaterally under the fourth gland of disease free, 4-week-old FVB/NJ female mice. Control VO-eGFP luciferase-expressing cells were injected as a control to monitor the estimated tumor growth. Palpable primary tumors were detected in all mice within 3–4 weeks post-injection. All animals developed primary tumors. Metastatic cell populations were identified by harvesting and processing the organs as described above via FACS analysis. Cancer cells were isolated using cell surface expression of CD44 and EpCAM. The experiment was performed in 4 different biological replicates.

### PCR analysis

gDNA was isolated from all MMTV-PyMT cell lines and control samples using a Zymo (California, USA) quick-DNA miniprep kit (Cat #: 11-317AC). Ear clippings from PyMT-positive male and female mice (courtesy of the Kessenbrock laboratory, UCI) were used as positive controls. gDNA samples isolated from 4T1 (ATCC CRL-2539) cell lines were used as negative controls. PCR amplification conditions and PyMT antigen detection were completed using the standard Jackson Labs genotyping protocol [28].

### Crystal violet proliferation assay

MMTV-PyMT cell lines were plated (5,000 cells/100 μL) in 96-well plates and incubated for 24 h. Cells were fixed in ice-cold methanol for 30 min. Cells were stained with a solution of 0.05% crystal violet in PBS for 30 min. The samples were then washed three times with PBS to remove excess dye and allowed to dry for 16–24 h. Crystal violet was recovered from cells via treatment with methanol, and the absorbance of the solutions at 595 nm was measured using a Gen5 microplate reader.

### Immunoblotting

Cells were lysed in RIPA buffer containing protease (Thermo Fisher Scientific, Cat #88,265) and phosphatase inhibitors (Sigma, Cat #4,906,845,001). Protein concentrations were measured using a BCA protein assay (Thermo Scientific, Cat #23,223). Samples were prepared in 2X SDS-PAGE loading buffer (containing 4% βME) and heated at 95 °C for 10 min. Samples were then separated on 4–20% polyacrylamide gels (BioRad) and transferred to nitrocellulose membrane (0.2 µm, BioRad). Membranes were incubated with blocking buffer (5% BSA in TBS containing 0.1% Tween-20®, TBST) for 1 h at room temperature. Blots were incubated in primary antibodies (Cell Signaling; 1:1000 dilution in blocking buffer) overnight at 4 °C. Blots were washed three times with TBST and then incubated with IRDye-conjugated secondary antibodies (LI-COR Biosciences; 1:10,000 dilution in blocking buffer) for 1 h at room temperature. Membranes were washed three times with TBST and imaged using a LI-COR Odyssey CLx imaging system.

### Bulk-RNA-seq

For each tissue-derived cell line, total RNA was extracted using the QIAGEN RNeasy kit with 2 replicates per sample. Sample replicates were distinct clonal isolates harvested from different MMTV-PyMT tumor-bearing mice. A modified SMART-seq2 protocol was used to generate cDNA and Nextera XT DNA Sample Prep Kit to build Illumina libraries. Samples were sequenced on a NextSeq500 with a min depth of 10 M reads. Raw reads were aligned to the mm10 genome with STAR [29], and quantification was performed using the GENCODE v21 annotation of the mouse genome using RSEM [30]. Count matrices for differential expression analysis were used as input for EdgeR [31]. An exact test was used for calling differential expressed genes with logFC > 2 and a p value < 0.05. EnrichR and metascape were used for gene ontology analysis.

### Single-cell RNA-seq methods

As with bulk RNA-seq analysis, scRNA-seq was performed on select tissue-derived cell lines with two replicates per sample. Replicates were distinct clonal isolates harvested from different MMTV-PyMT tumor-bearing mice. Cell lines that originated from lung with CD44^high/low^ signatures were identified as the samples with most transcriptional changes and were selected for single-cell analysis, along with a lymph node high sample. Single-cell suspensions from these tissues were used as input for the ddSeq platform and cDNA synthesis and library prep was done following the SureCell™ Whole Transcriptome Analysis 3' Library Prep Kit. The bioinformatic pipeline included Ddseeker [31], a custom demultiplexing script to generate individual fastq, while kallisto [32] was used to quantify the transcripts in our sample using the mm10 and annotation GENCODE v21. Single-cell analysis was done using Seurat v3.2.3 [32]. Cells with more than 250 genes and less than 10% mitochondrial reads were used for the analysis. Monocle2 [32] was used to infer pseudo-time progression. Min. read depth 34 M.

### Data availability

Fastq files for bulk and single-cell datasets as well as their corresponding processed matrices are available in GEO (Accession Number: GSE165393).

## Results

### Generation of breast cancer cell lines to examine tumor heterogeneity and metastatic disease

**Fig. 1:**
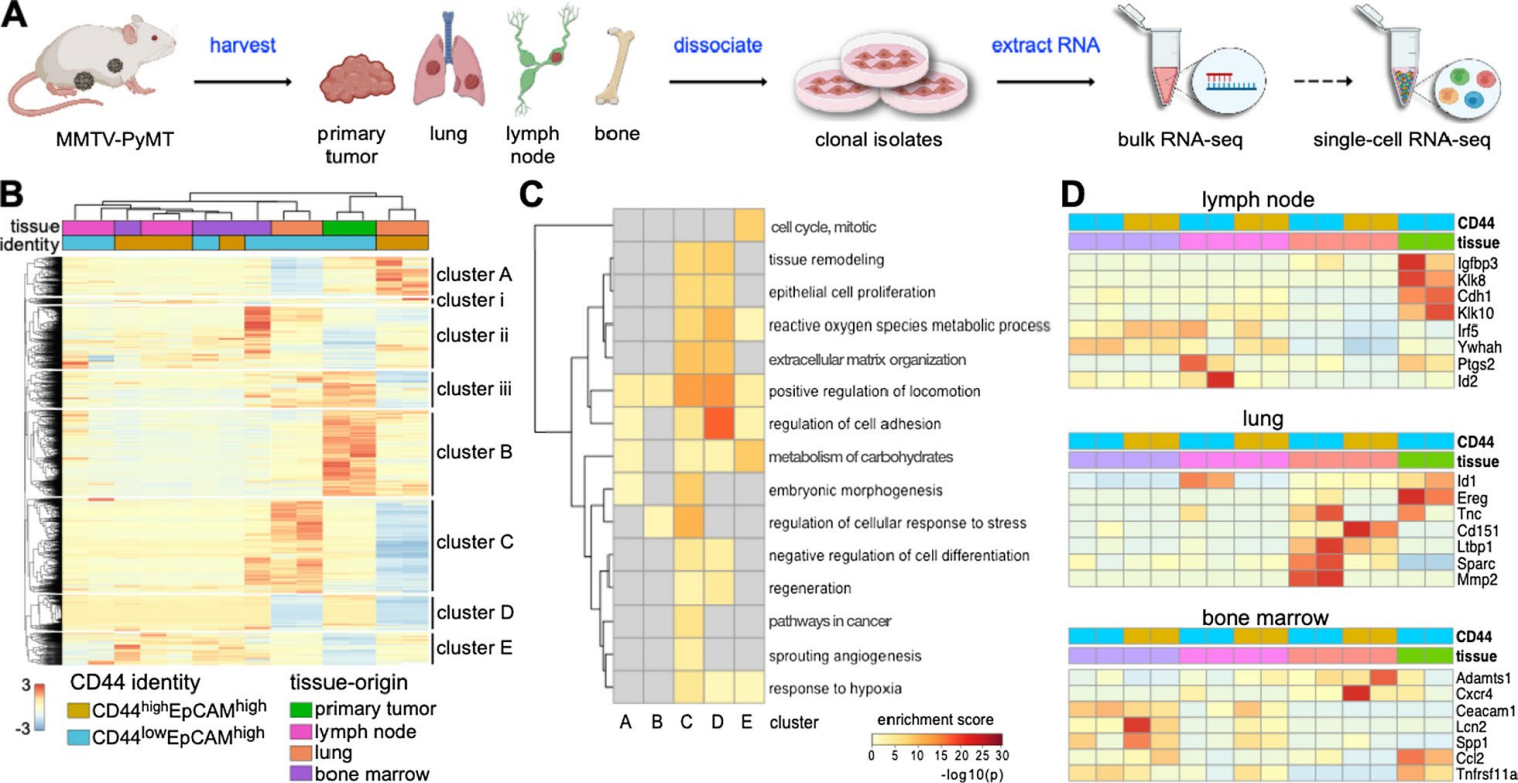
Clonal isolates from MMTV-PyMT breast cancer model exhibit distinct gene expression patterns. **A** Overview of cell isolation procedures and gene expression analyses. Tumors were harvested from mice and single cell suspensions were prepared. Cells were sorted based on CD44 and EpCAM expression. RNA was extracted for transcriptome profiling. Select samples were further analyzed via single-cell RNA-seq. **B** Heat map of DEGs from tissue-specific metastatic cell lines and primary tumor sample. Expression levels for 5509 unique genes are shown. Values were normalized by row, and hierarchical clustering was used to sort the transcripts. Columns were clustered based on the tissue origin and CD44 expression level for each sample. Eight distinct gene clusters were observed, with clusters of interest annotated **A**–**E**. **C** GO-term enrichment analysis of clusters **A**–**E** from (**B**). GO terms were used to identify ontologies and biological processes relevant to cancer metastasis. Terms were also analyzed for signatures specific to the tissue of origin. The heat maps indicate the relative enrichment of the pathways across each cluster (columns). **D** Bulk RNA analysis revealed distinct gene expression patterns relevant to organ tropism. A panel of markers associated with tissue-tropic breast cancer metastases was examined across all samples. Clusters were assigned based on the tissue origin and CD44 expression level for each sample

To gain insight into breast cancer heterogeneity, we derived a suite of tissue-specific metastatic cell lines from MMTV-PyMT mouse tumors (Fig. 1A). Tumors were harvested from the mammary fat pad (MFP) and tissues harboring distal metastases, including lymph nodes (LN), bone marrow (BM), and lungs (L). Samples were processed into single cell suspensions and further expanded. The organ-derived cultures were subjected to conditions that favored cancer cell outgrowth in vitro. Cells were ultimately sorted based on CD44 and EpCAM expression [27] to remove fibroblasts from the samples. CD44 is routinely used as a marker of aggressive metastatic breast cancer [33]. FACS sorting provided two populations: CD44^low^/EpCAM^high^ and CD44^high^/EpCAM^high^ (Additional file 1: Fig. 1A-B). PCR was also used to confirm the presence of the PyMT viral antigen in the cell isolates (Additional file 1: Fig. 1C). For the sorting and PCR assays, an established MMTV-PyMT cancer cell line (VO) and a common fibroblast cell line (3T3) were used as positive and negative controls, respectively. The tumorigenicity and metastatic propensity of the sorted MFP cell line was validated in vivo by injecting cultured cells into wild-type female FVB mice (Additional file 1: Fig. 1B). The presence of metastatic tumors was confirmed by harvesting LN, BM, and lung tissue from the re-injected mice. Single cell suspensions were formed and flow cytometry analysis confirmed the presence of CD44^low^/EpCAM^high^ and CD44^high^/EpCAM^high^ cells in the harvested tissues.

### Cancer cell lines exhibit distinct gene expression changes relative to metastatic progression

We used the tissue-derived cell lines to investigate transcriptional changes that occur during breast cancer metastasis. RNA was extracted from all cell samples, and transcripts for established breast cancer genes were identified (Additional file 1: Fig. 2) [34–36]. Hierarchical clustering was performed on 5,509 DEGs. Eight distinct gene clusters (A-E; i-iii) were observed, as shown in Fig. 1B. The transcripts were organized based on CD44 expression (CD44^high^/EpCAM^high^ or CD44^low^/EpCAM^high^) and tissue origin (primary tumor, lymph node, lung, or bone marrow). We focused on the five most prominent gene clusters (A-E) relevant to cancer progression for further analysis. Compared to the primary MFP tumor, the tissue-derived samples exhibited distinct upregulated and downregulated genes. Highly upregulated genes in MFP cells localized to cluster B. Lung-derived samples (CD44^low^ and CD44^high^) shared some similar transcriptomic changes (clusters D, E), but they also showed DEGs unique to their CD44 identity (clusters A, C). LN and BM samples trended similarly with MFP tumor cells, showing moderate expression of genes in cluster C.

To understand the biological relevance of the DEGs relevant to each cluster, we performed pathway enrichment analysis. Heat maps of the top 100 significant pathways revealed a multitude of cellular and molecular processes associated with cancer (Additional file 1: Fig. 3, Additional file 2). Pathways specifically relevant to cancer metastasis are shown in Fig. 1C, along with the corresponding enrichment score for each cluster. The upregulated genes for CD44^low^/EpCAM^high^ lung-derived cells in cluster C correlated with embryonic morphogenesis and hypoxia response pathways. Both of these pathways are critical to cancer cell growth in hostile environments [37, 38]. Some of the specific transcripts observed included those from the well-established cancer survival genes *ALDH1A1, SURVIVIN, XIAP, HSPG2, BCL9,* and *SOX4* [39, 40]. Interestingly, these same genes were downregulated in CD44^high^/EpCAM^high^ lung-derived cells (Additional file 2). Cluster A was enriched in regulatory pathways associated with cell adhesion, correlating with the expression of *DDR1, HOXA7, MMP2, THBS1, TNFRSF14,* and *TGFB2* [41, 42]. Cluster A was also enriched in pathways associated with cellular locomotion, corroborated by the expression of *SERPINE1, PDGFA, ITGAV,* and *ITGB1BP1* [43, 44]. Both sets of upregulated genes were observed in CD44^high^/EpCAM^high^ lung-derived cells, but not MFP-derived or CD44^low^/EpCAM^high^ lung-derived cells. CD44^high^/EpCAM^high^ lung-derived cells also exhibited upregulated carbohydrate metabolism genes, pathways enriched in clusters A and E. Cluster E also correlated with other upregulated metabolic genes (*PFKFB3, SDC3,* and *GPC3*) in both lung-derived cell lines. These same genes were downregulated in the MFP cells [45, 46].

Metastatic breast cancer cells are known to preferentially colonize specific organs, a process known as organotropism [47]. The cross-talk between metastatic cells and the distal microenvironment leads to the formation of the pre-metastatic niche, which can influence cancer cell homing [48, 49]. We examined whether the clonal isolates recapitulated features of organotropic metastases to lymph nodes, lung, and bone (Fig. 1D). Gratifyingly, we identified gene expression patterns that differed among the metastatic cell types based on their tissue of origin. LN-derived cells lines expressed genes relevant to metastatic lymphatic niches (e.g., *IRF5, YWHAH, PTGS2,* Fig. 1D) [50–55]. MFP-associated genes (*CDH1* and *IGFBP3* [50–55]) were also observed in the LN-derived lines, albeit to a lesser extent. In the case of the lung-derived cells, lung-tropic genes (e.g., *SPARC, MMP2, LTBP1, ID1*, and *CD151*) associated with high metastatic propensities [50–55] were upregulated. *EREG*, a marker expressed by cells in the mammary gland [50–55], was downregulated in the lung-derived cells. BM-derived cells expressed genes associated with BM metastases, including *CEACAM1* and *LCN2* [50–55]. Expression of *CCL2, ADAMST1*, and *CXCR4* was also observed in BM-derived cells albeit to a lesser extent. The expression of a select set of markers was further confirmed by Western blot analysis (Additional file 1: Fig. 4). Collectively, these transcriptome changes could contribute to sub-clonal evolution during cancer progression across the different metastatic niches.

We also compared the gene expression changes among the metastatic cells and to those from the primary tumor. Overall, we observed that samples derived from organs further away from the primary tumors had greater numbers of DEGs, regardless of the CD44 designation (Additional files 3–8). Lung-derived samples exhibited the most DEGs (4,411 genes in total) compared to cells derived from the lymph node (1753 genes in total) or bone marrow (2,985 genes in total, Additional file 1: Fig. 5). Volcano plots of DEGs from each tissue-derived metastatic cell line (CD44^high/low^/EpCAM^high^) compared to the primary tumor sample revealed genes involved in metastatic progression (Additional file 1: Fig. 6 and Additional files 3–8). Interestingly, some of the greatest differential expressions observed involved organotropism-associated genes (*MMP2* and *EREG*) identified in Fig. 1D.

**Fig. 2:**
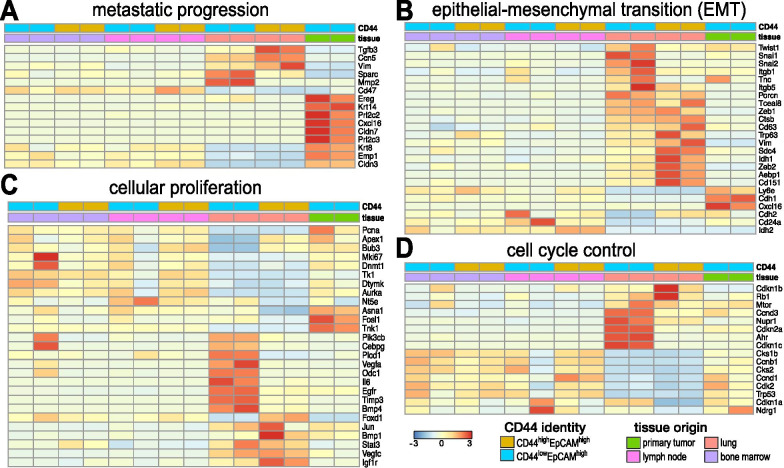
Cancer cell lines exhibit distinct gene expression patterns relative to metastatic disease progression. Bulk RNA analysis revealed differential gene expression patterns relevant to **A** metastatic progression, **B** epithelial–mesenchymal transition (EMT), **C** cellular proliferation, and **D** cell cycle control among the tissue-derived isolates and primary tumor. Columns were clustered based on CD44 expression and tissue origin as indicated. Select genes relevant to metastatic progression are displayed in the heat map

We aimed to further characterize the metastatic cell lines via GO-term enrichment analysis. To this end, we examined gene expression changes relevant to metastatic progression, epithelial-to-mesenchymal transition (EMT), cellular proliferation, and cell cycle control (Fig. 2). In the case of metastatic progression, we observed that MFP samples expressed high levels of classical markers associated with pre-metastatic lesions (e.g., *EREG, KRT14, CLDN7, KRT8, EMP1,* and *CLDN3,* Fig. 2A) [48, 49]. These markers were decreased in cells from distal metastatic sites (e.g., LN- and lung-derived cells). LN- and lung-derived cells, by contrast, exhibited upregulated levels of mesenchymal markers (e.g., *CCN5, ZEB1, VIM, SPARC*, and *TGFB3*) [13, 56–59]. Expression levels were highest in CD44^high^/EpCAM^high^ lung-derived cells. Lung-derived cell lines also showed increased expression of well-established EMT markers (*SNAI1/2* and *CD63*) [56] and markers associated with poor prognosis in patients (*FOXC1*, *AEBP1, SDC4*, and *IDH1*, Fig. 2B) [52, 60]. Interestingly, *SNAI1/2* and *CD63* expression were highest in CD44^low^/EpCAM^high^ lung-derived cells, while the poor prognosis indicators listed were higher in CD44^high^/EpCAM^high^ lung-derived cells. The upregulation of mesenchymal markers and downregulation of epithelial markers in lung-derived cells are indicative of cellular de-differentiation [52, 60–62], suggesting that the lung-derived cells recapitulate EMT.

EMT typically correlates with changes in cell proliferation and dysregulation of cell cycle control during cancer progression. These trends were apparent in the gene expression profiles for both CD44^high^ and CD44^low^ cells (Fig. 2C, D). As expected, the highly proliferative lung-derived CD44^high^ cells expressed low levels of growth arrest genes *CDKN1A* and *CDKN2A* (Fig. 2D) [37, 38]. Interestingly, we observed a stark difference in gene expression for lung-derived CD44^low^ cells. Although these cells expressed genes relevant to cellular proliferation and angiogenesis, they exhibited upregulated levels of the growth arrest genes (Fig. 2D). Growth arrest signals could dampen the expression of other genes that are master regulators of downstream cell function. One such gene, mTOR, was expressed in the lung-derived CD44^low^ cells. The levels of mTOR were comparable to expression in CD44^high^ cells. From these results we postulate that lung CD44^low^ cells, albeit capable of cell division, are not dividing as rapidly as their CD44^high^ counterpart.

### Analyses of common biological pathways reveal intratumor heterogeneity

MMTV-PyMT has recently been used as a model to study the impacts of CD44 on metastases [55]. Ex vivo analysis of tumors from a single micro-metastatic site revealed two subgroups of cells with differential CD44 expression. CD44 expression correlated with altered gene expression relevant to EMT and MET and differential growth rates [52, 60]. Post-metastatic colonization, CD44 expression levels did not remain constant and were frequently switched between the subgroups. The fluid transition from EMT to MET phenotypes demonstrates how complex and context-dependent breast cancer can be. The morphological changes induced by CD44 expression also affected the tumor-initiating capabilities of the tumor cells. However, the critical regenerative CSC populations were found in both CD44-expressing groups, warranting further characterization. Similar spectrums of behavior have been documented in other studies [23, 55, 59].

**Fig. 3:**
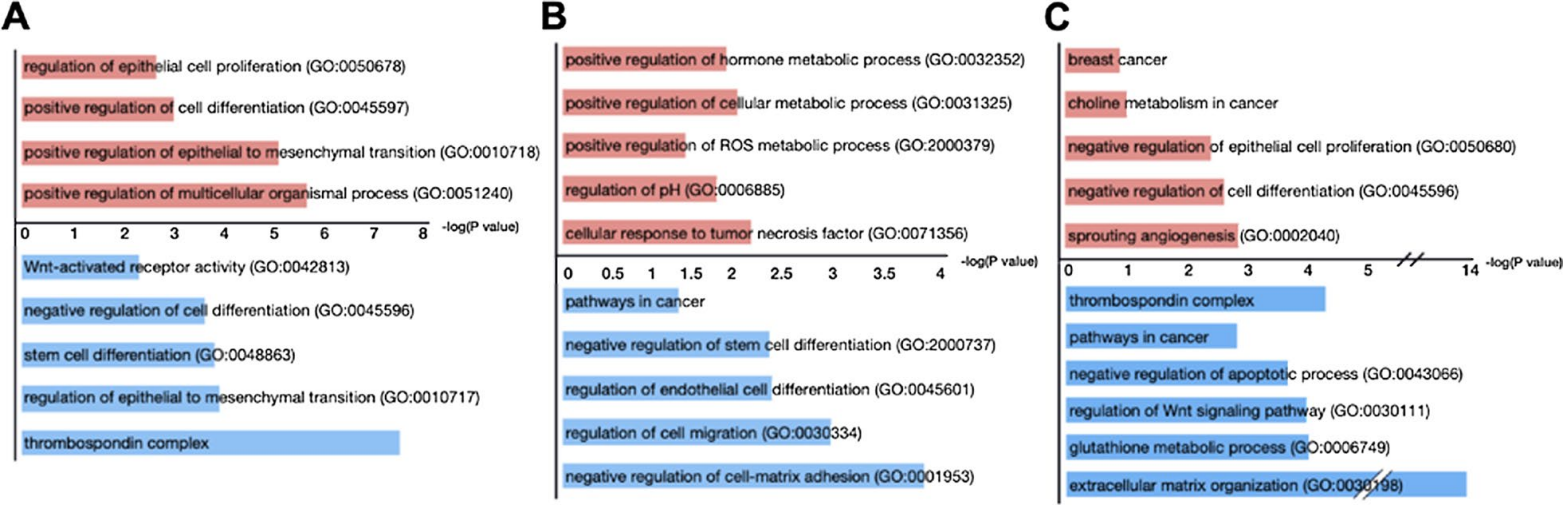
CD44 expression correlates with different transcriptome patterns in organ-derived cell lines. Differentially expressed genes for **A** CD44^high^ (red) and CD44^low^ (blue) EpCAM^high^ cells (across all samples) were used to generate GO terms. CD44^low^ cells exhibited high levels of expression for genes relevant to tumor microenvironment remodeling and tumor dormancy markers. CD44^high^ cells exhibited higher levels of gene expression associated with GO terms related to cellular proliferation, tumor aggression, and EMT. Differentially expressed genes and associated GO terms for CD44^high/low^ EpCAM^high^ cells from **B** lymph nodes and **C** lungs are also shown. For **A**–**C**, the − log(P value) scale applies to both the CD44^high^ (red) and CD44^low^ (blue) samples

To more closely examine the impacts of CD44 expression in our organ-derived cells, we analyzed DEGs based on CD44 levels (Additional files 9–11). We identified upregulated genes for CD44^low^/EpCAM^high^ (374 genes) and CD44^high^/EpCAM^high^ (276 genes) signatures across the suite of cells (Additional files 12–13). Some genes were shared across the tissue types. We also examined the GO terms and pathways associated with the DEGs from CD44^low^/EpCAM^high^ and CD44^high^/EpCAM^high^ samples. Clear differences were observed between the two CD44 signatures across the tissue-derived samples (Fig. 3A and Additional files 12–13). Cells with CD44^high^ signatures exhibited an increase in GO terms and associated genes related to cellular proliferation, tumor aggression, and EMT. Indeed, CD44^high^ cells from lung and lymph node samples were experimentally observed to exhibit increased proliferation rates compared to CD44^low^ cells (Additional file 1: Fig. 7). DEG analysis further revealed that CD44^low^ cells exhibited higher gene expression levels relevant to tumor microenvironment remodeling and stem cell markers. Interestingly, CD44^low^ signatures also correlated with signaling pathways known to be important for stem cell maintenance and Wnt-activated receptor activity. CD44^low^ signatures were further negatively correlated with cell differentiation pathways, supporting the idea of retained cellular dedifferentiation. The thrombospondin complex pathway, key in the maintenance of cancer stem cell dormancy in breast cancer, was also present in the CD44^low^ signature [16, 61, 63].

We further examined the heterogeneity of CD44^high^ versus CD44^low^ expression within single tumors. Volcano plots revealed DEGs in CD44^high^/EpCAM^high^ versus CD44^low^/EpCAM^high^ from lymph node-derived, lung-derived, and bone marrow-derived metastatic clonal isolates (Additional file 1: Fig. 8A-C). The DEGs for these samples were also subjected to pathway enrichment analysis (Fig. 3B, C, Additional files 14–17). DEGs upregulated in lymph node-derived CD44^high^/EpCAM^high^ cells correlated with cellular metabolism and pH regulation (Fig. 3B) observed in aggressive cancer phenotypes. Similar pathways were not observed in the corresponding CD44^low^/EpCAM^high^ lymph node-derived cells. The DEGs for these cells, by contrast, were enriched in pathways regulating stem cell differentiation, cellular migration, and cell–matrix adhesion (Fig. 3B). For the lung-derived samples, the CD44^high^/EpCAM^high^ cells exhibited upregulated DEGs relevant to cancer metabolism, cellular proliferation, and angiogenesis (Fig. 3C). The DEGs for the corresponding CD44^low^/ EpCAM^high^ lung-derived cells were also enriched for pathways relevant to cancer progression and cellular metabolism, in addition to the thrombospondin complex and extracellular communication (Fig. 3C). Similar analyses were performed with bone marrow-derived cells to reveal unique GO terms and transcriptomic changes (Additional file 1: Fig. 8D-E). Upregulated DEGs for BM-derived CD44^low^/ EpCAM^high^ cells were mainly associated with immune and cytokine activity.

**Fig. 4:**
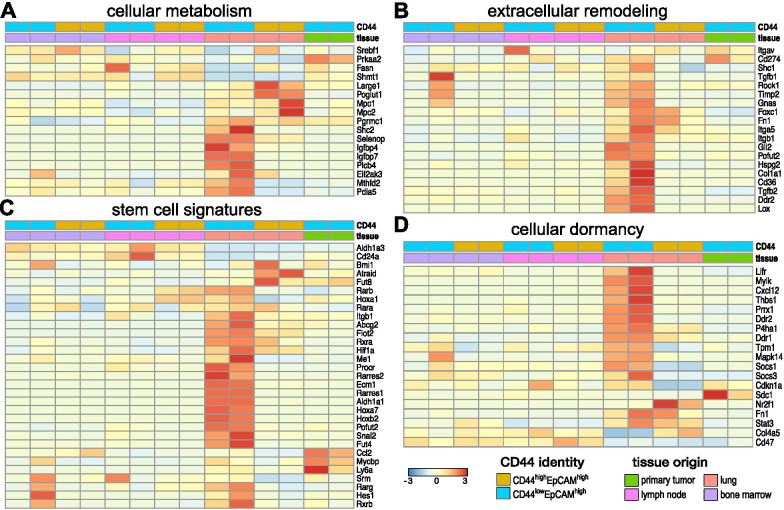
Intratumoral heterogeneity observed across organ-derived cell lines. Bulk RNA analysis of the organ-derived cell lines revealed distinct expression patterns relevant to **A** cellular metabolism, **B** extracellular remodeling of the microenvironment, **C** stem cell signatures, and **D** cellular dormancy among the cell lines. Clusters were assigned based on CD44 expression and the metastatic origin of each cell line as shown in the lower right

Based on our DEG analyses, we examined additional known markers of breast cancer metabolism and extracellular remodeling across the entire set of organ-derived cell lines [64, 65]. We observed increased levels of cellular metabolism markers (e.g., *PLCB4, IGFBP7, IGFBP4, SHC2, PGRAMC1, MTHFD2*) specific to lung-derived CD44^low^ cells (Fig. 4A). Lung-derived CD44^high^ cells exhibited higher levels of *MPC1, MPC2, POGLUT1*, and *LARGE1* expression. Interestingly, we did not observe upregulation of other cancer-related drivers of energy consumption in either of the lung-derived cell lines compared to MFP-derived samples [66]. Extracellular remodeling has been shown to improve cancer colonization and perpetuate dedifferentiated stem-like cellular states [61, 62]. We identified markers relevant to extracellular remodeling known to promote the survival of metastatic lesions (*CD36, CD274, FOXC1*) in the lung-derived metastatic cells (Fig. 4B). The expression levels were noticeably enhanced in these samples compared to the MFP tumor. Lung-derived CD44^low^ cells also exhibited upregulated levels of genes associated with mesenchymal cells and more dedifferentiated phenotypes in advanced cancers (e.g., *FN1* and *POFUT2*) [13].

### CD44^low^ cell lines exhibit classic signatures of stem cells

As noted earlier, the lung-derived CD44^low^ cells exhibited reduced levels of some markers of cellular proliferation and division (Fig. 2C, D). These cells also expressed genes known to be important for CSC survival and function (Fig. 4B) [67]. Changes in gene expression relevant to matrix remodeling have been known to sustain CSCs in a functional, but dormant, non-dividing state. To further examine whether the lung-derived CD44^low^ cells harbored CSC properties, we evaluated a panel of known breast cancer stem cell markers across our suite of metastatic isolates. Comparisons were made to the known cellular differentiation marker CD24 [68, 69]. As shown in Fig. 4C, lung-derived CD44^low^/EpCAM^high^ cells exhibited an increase in the stem cell-associated markers and a decrease in CD24 expression. Furthermore, expression of *ALDH1A1*, a breast cancer-specific stem cell marker associated with resistance to some chemotherapies, was elevated [68, 69]. CSCs can endure some drug treatments and survive in metastatic environments due, in part, to their ability to modulate their metabolism and compensate for oxidative stress [67, 68]. The lung-derived CD44^low^ cells exhibited gene expression profiles consistent with these phenotypes, including the upregulation of retinoic acid (RA) pathway (*RARA/B/G, RXRA/B, RARRES1/2*) essential for cell survival. Opposite trends were observed for lung-derived CD44^high^ cells. These cells expressed higher levels of genes associated with cell growth and aggressive metastases.

We further examined the entire suite of cells lines, for markers of breast cancer dormancy (Fig. 4D). The lung-derived CD44^low^ cells expressed higher levels of genes associated with the thrombospondin complex, a well-known dormancy marker in breast cancer [16, 70]. Additional markers, including *MAPK14*, *DDR1/2,* and *MYLK*, were also observed among this cell population. Collectively, these data suggest that lung-derived CD44^low^ cells express CSC-relevant genes that can maintain cells in a dormant or low proliferative state. CSC identification remains challenging, though, owing to difficulties in isolation [19–21].

### Single cell RNA-seq reveals distinct clusters relevant to metastatic progression and intratumor heterogeneity

**Fig. 5:**
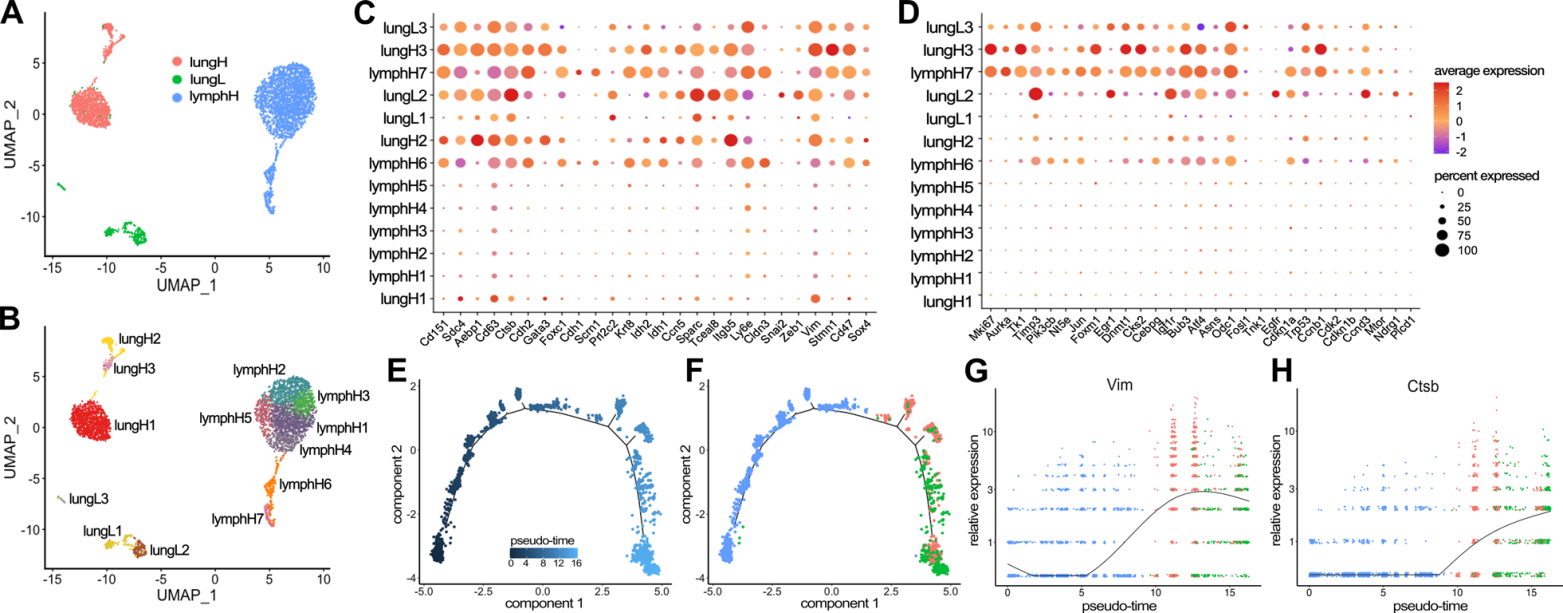
Single-cell RNA-seq revealed tissue-specific clusters and heterogeneity during metastatic disease progression. **A** Clustering of 4124 cells that passed filtering. Tissue-specific clustering was observed for lungH, lungL, and lymphH cells. **B** Thirteen clusters were recovered based on a combination of tissue origin and CD44 signature. **C**, **D** Gene expression profiles for select cancer-relevant genes (columns) relevant to **C** EMT and **D** cell proliferation among the thirteen clusters (rows). The size of each dot represents the percentage a specific gene is expressed compared to all other transcripts. The color gradient of the dot indicates the average expression of the gene. **E** Monocle2 pseudo-time analysis was performed, and the metastatic trajectory of distinct cell clusters is shown. The color gradient indicates pseudo-time progression. **F** Tissues of origin indicated on the pseudo-time map (as in **A**). Changes in EMT-relevant genes (from **C**) were probed across pseudo-time and tissue of origin. **G** The expression of *VIM*, an EMT marker, increased over lymph node populations with maximum expression in lung high populations. This analysis was repeated for **H** *CTSB*, a marker of invasion, where the low expression across lymph node populations increased in expression across lung high and lung low populations

To further validate that our isolated cell lines capture the intratumoral heterogeneity observed during de novo disease progression, we performed scRNA-seq on a subset of metastatic samples. Based on the amount of differential gene expression observed in bulk RNA-seq, we chose lung-derived CD44^low^ (lungL), lung-derived CD44^high^ (lungH), and LN-derived CD44^high^ (lymphH) cells for the analyses. In all, 4124 cells were used in the clustering analyses: lungL (334 cells), lungH (1085 cells), lymphH (1694 cells). Clusters were visualized using UMAP (Fig. 5A). Clusters for each of the three cell types—lungL (green), lungH (pink), and lymphH (blue)—were identified.

We further examined the heterogeneity of the samples using hierarchical clustering (Additional file 1: Fig. 9). The UMAP plot depicted 13 distinct cell clusters from the three respective cell samples (Fig. 5B). LymphH cells had the largest number of subclusters (7), with clusters 1–5 being very similar in composition (Additional file 1: Fig. 9). Clusters 6 and 7 (lymphH6 and lymphH7) exhibited higher levels of pro-survival/cell cycle regulation genes (*BIRC5, TOP2A, CENPF*) associated with metabolically active cancer pathways. We observed the lung CD44^high^ (lungH) cells divided into three different clusters. The majority were localized to cluster lungH1 and expressed lower levels of metastatic cancer cell markers (*ALDH3A1, VIM, TCEAL9*). Similar metastatic cancer markers were upregulated in cluster lungH2. This subpopulation further displayed upregulated markers associated with the microenvironment and EMT (*AEBP1, ITGB5, FN1*).

### EMT and proliferation markers show distinct tissue-specific gene expression

We identified transcriptomic changes relevant to cancer metastasis that drove the designation of each cluster. Guided by the bulk RNA-seq results, we examined the expression of EMT and metastatic progression markers (Fig. 5C). We used dot plots to visualize the average expression of each gene and the percentage it was expressed in the sample set. As expected for progressive disease, we observed genes associated with primary tumors and low metastatic lesions (*CLDN3, KRT8, CDH1, CDH2*) primarily in the lymphH populations. Expression of these genes diminished at more distal sites (lung-derived samples). Conversely we observed genes associated with aggressive metastatic disease and EMT (*VIM, SPARC, ZEB1, SNAI2, CTSB*) upregulated in lung populations compared to lymph nodes. Changes in the expression of breast cancer marker CD63 were also observed during disease progression. Interestingly we observed similar gene expression changes for EMT in clusters lymphH6, lymphH7, and lungH3.

To further examine the changes in gene expression, we performed pseudo-time analysis of the single cells using Monocle2. The metastatic trajectory of the cells showed distinct clusters across the pseudo-time (Fig. 5E). We identified the composition of the cells by coloring the pseudo-time map with the tissues of origin (Fig. 5F). We observed that the majority of cells at the beginning of the pseudo-time (0) are lymphH-derived with a few lungL cells. Lung-derived cells became prominent further on the graph around pseudo-time 8. Interestingly, we observed lungL cells present across pseudo-time that cluster heavily toward the end of the graph (pseudo-time 12–16). We probed for changes in *VIM* and other EMT-relevant genes across pseudo-time and tissue of origin. As we previously observed in Fig. 5A, *VIM* expression increases over lymph node populations (Fig. 5G). *VIM* expression is highest in lung high populations and drops down in lung low populations. *CTSB*, a marker of invasion, showed relatively low expression across lymph node populations (Fig. 5H), but higher expression along pseudo-time in lungH and lungL populations.

Cellular proliferation markers were also analyzed via scRNA-seq (Fig. 5D). Increases in proliferative markers (*AURKA, MKI67*) were observed for clusters lymphH6 and lymphH7, with the greatest expression in lungH3. We also observed many similarities between clusters lymphH7 and lungH3 with regard to EMT- and proliferation-associated gene expression. These correlations could potentially signify the metastatic progression of the disease from the lymph node (lymphH7) to lung (lungH3). LungL clusters, by contrast, expressed lower levels of genes involved in proliferation. These clusters expressed higher levels of genes involved in cell cycle control and growth inhibition (*CDKN1A, CDKN1b, CCND3*). We further probed for changes in a panel of proliferation-associated genes across pseudo-time and tissue of origin using Monocle2 (Additional file 1: Fig. 10).

### Cellular metabolism and extracellular remodeling markers show distinct tissue-specific changes

**Fig. 6:**
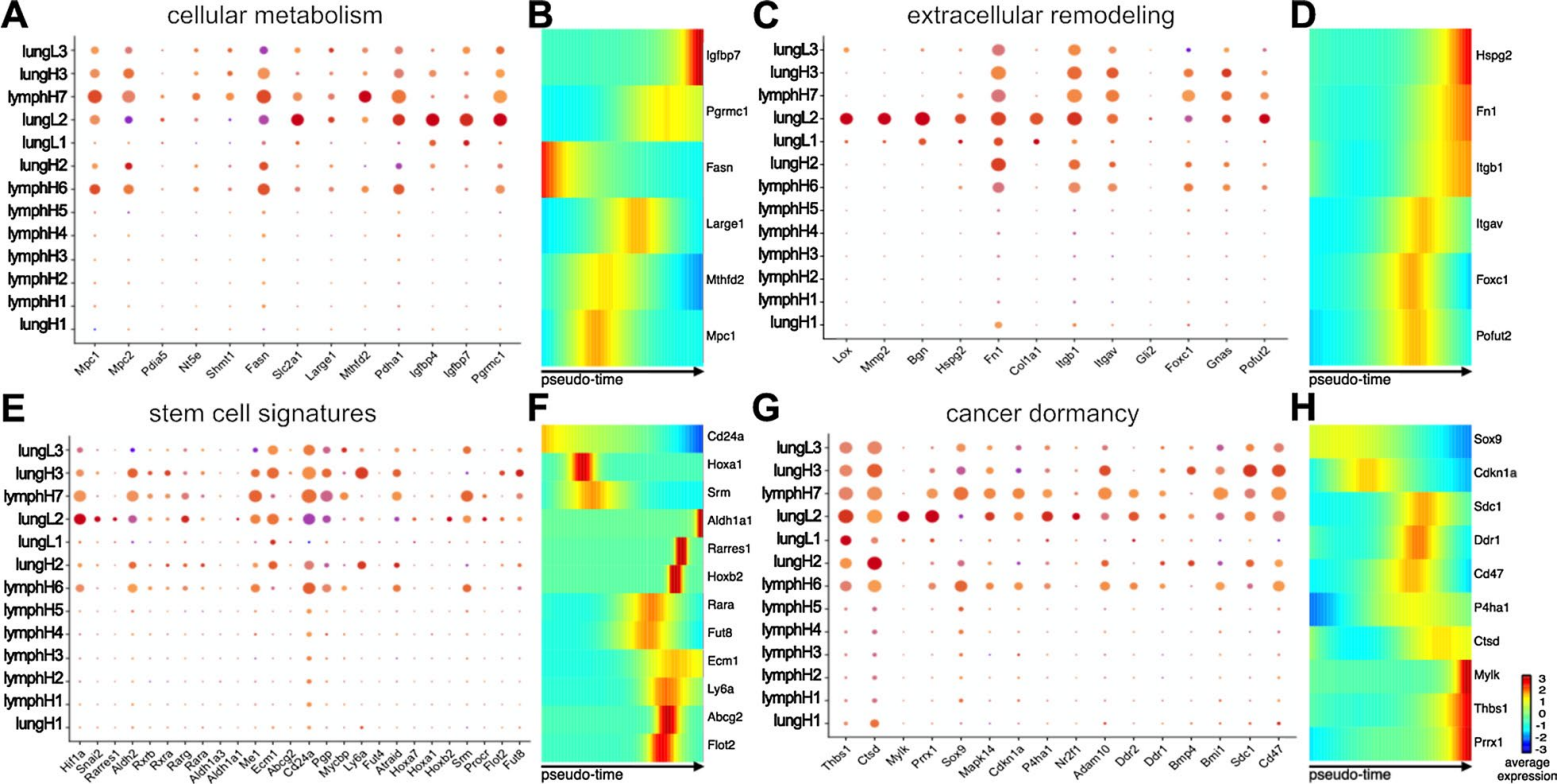
Single cell profiling revealed tissue-specific changes. **A** Dot plot analysis of cancer metabolic markers relevant to cancer progression for select clusters guided by the bulk RNA seq data. **B** Heat maps of differentially expressed genes relevant to cancer metabolism were identified across the pseudo-time using Monocle2. The color gradient indicates the average expression across the pseudo-time, trending from dark blue to red. Similar dot plot analyses and pseudo-time heat maps are shown for markers relevant to **C**–**D** tumor microenvironment remodeling, **E**–**F** stem cell signatures, and **G**–**H** cell dormancy

We identified gene expression markers relevant to cellular metabolism and extracellular remodeling that drove the formation of each cluster. For example, lungH3 cells expressed upregulated levels of genes associated with cell cycle and intracellular metabolism (*TOP2A, CENPF, HTRA1, STMN1*) (Additional file 1: Fig. 9). These data suggested that cells within the lungH3 cluster exhibit the highest metastatic propensity of the lung subsets. We further observed an increase in insulin-like growth factors (*IGBFP4, IGBFP7*) across different lung-derived clusters, specifically in the lungL2 cluster (Fig. 6A). Interestingly, the expression of *PDHA1*, a critical component for pyruvate to acetyl-CoA conversion, was primarily upregulated in lungL2, lymphH7, and lymphH6 clusters. We probed for changes in these metabolism-related genes across pseudo-time and tissue of origin using Monocle2. As we previously observed in Fig. 6A, *IGBFP7* expression was significantly upregulated in lungL single cell populations as pseudo-time progressed (Fig. 6B).

Guided by bulk RNA-seq results, we further examined extracellular remodeling markers using scRNA-seq (Fig. 6C and Additional file 1: Fig. 9). We observed the most dynamic expression of extracellular remodeling-associated genes in the three lungL clusters. The lungL1 cluster had distinct differences in gene expression relevant to extracellular matrix interactions (*MGP, BGN, CCN2, FN1, ITGB1*). LungL2 cells expressed similar genes, along with upregulated genes associated with immunosuppressive proteins (*SLPI*) and embryonic glandular hormone (*PRL2C3*). However, cells in the lungL3 cluster lacked significant expression of extracellular remodeling genes that were present in lungL1 and lungL2 cells. We investigated changes in extracellular remodeling-relevant genes (*FN1, ITGB1*) across pseudo-time and tissue of origin using Monocle2 (Fig. 6D). *FN1* had relatively low expression across lymph node populations, but increased expression was observed in lungH and lungL cells populations starting at pseudo-time 10. *ITGB1* was prominently expressed across most lungH, lungL, and some lymphH cell clusters (Fig. 6C). However, pseudo-time analysis of the single cells only attributed a significant upregulation of *ITGB1* expression in lungL cells at the end of the pseudo-time (Fig. 6D).

### CD44^low^ lung-derived cell lines harbor markers related to cancer stem cells and tumor dormancy

Although CD44 expression has traditionally been used as a marker of metastatic disease, recent publications have demonstrated that cellular expression of CD44 fluctuates during cancer progression [55]. This has prompted other markers to be used in parallel with CD44 when examining the metastatic potency and cancer-initiation capability of cells. Toward this end, we used scRNA-seq to probe for markers of CSC and tumor initiation in the breast cancer model (Fig. 6E). We observed an increase in stem cell-associated genes (*ALDH1A1, ECM1, ME1, ABCG2,* and *SNAI2*) with a subsequent decrease in differentiation marker, *CD24A*, in lungL1 and lungL2 clusters. Interestingly, lungL2 had the greatest upregulation in *HIF1A*, a known marker of hypoxia and poor prognosis in breast cancer. We previously observed that cells in the lungL3 cluster separate from the other two lungL clusters (Fig. 5B). These cells have unique changes in gene expression relevant to embryonic and cancer stem cells (*KDEL1*, *PRL2C3*, *EBP1*, Additional file 1: Fig. 9). Similar to clusters lungL2 and lungL3, stem cell genes *ALDH1A1*, *ABCG2*, and *SNAI2* were upregulated in lungL3 cells. However, other stem cell-associated genes (*ME1*, *HIF1A*) were downregulated. Additionally, lungL3 cells displayed an increase in *CD24A*, a marker of cellular proliferation and decreased stemness. Similar to the bulk RNA sequencing data, we saw an increase in *ALDH1A1* expression and stem cell survival metabolomic markers from the RA family in lungL2 and lungL3 clusters that were not present in lungL1 cells. In contrast, CD44^high^ clusters lungH3, lungH2, lymphH6, and lymphH7 exhibited an increase in genes regulating growth and invasion (*ECM1*, *CD24*, *FUT8*). While these CD44^high^ clusters showed a downregulation in stem cell markers *ALDH1A1* and *ABCG2*, they did show an increase in *ALDH2*, a stem cell marker less common in breast cancer.

We examined the changes in genes associated with cellular differentiation and stem cell capabilities (*CD24A*, *ALDH2*) using Monocle2 (Fig. 6F). *CD24A* expression was upregulated in lymph node populations and then downregulated as pseudo-time moved into the lung populations. Upregulation of stem cell marker *ALDH2* was observed across a select amount of cells for all cell types across pseudo-time (Additional file 1: Fig. 10).

Cancer dormancy is a formidable obstacle in breast cancer research and treatment [71]. Previous work has shown that some dormant breast cancer cells have similar genomic profiles as CSCs [33, 72]. We examined whether our cell lines expressed markers of cancer dormancy and cancer stem cell-associated markers. Similarly to the bulk RNA-seq analyses, we saw an increase in genes regulating cell proliferation and increasing tumor dormancy in lungL2 cells and, to a lesser extent, lungL1 cells (Fig. 6G). This pattern was not sustained in the lungL3 subcluster. Instead, lungL3 cells showed a decrease in dormancy-associated genes *FN1*, *CD47,* and *THBS1*. Interestingly, lungL3, lungL2, and lungH3 clusters showed increased expression of breast cancer dormancy cell-associated maker *SDC1* [73, 74]. Master regulator of morphogenesis, *SOX9* [75], was upregulated in lungL3, lymphH7, and lymphH6 subclusters. We further probed the changes in cancer dormancy-relevant genes *CTSD*, *THBS1* across pseudo-time, and tissue of origin using Monocle2. *CTSD* expression fluctuated across lymph node populations (Fig. 6H). The expression of *CTSD* was upregulated through lungH populations and plateaued in the lungL populations across pseudo-time. *THBS1* exponentially upregulated expression at pseudo-time 10 in lung high and lung low populations (Additional file 1: Fig. 10).

## Discussion

**Fig. 7:**
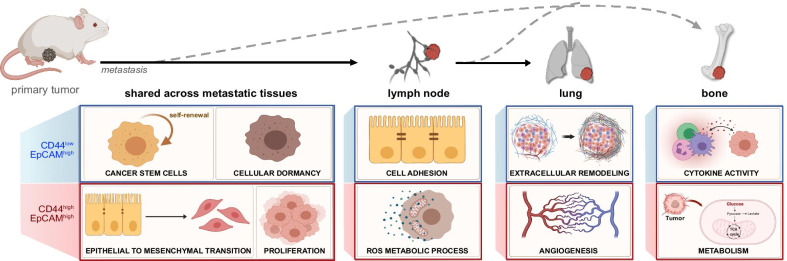
Summary of key findings from transcriptomic analysis of MMTV-PyMT-derived cell lines

Breast cancer comprises cell subpopulations that are genetically and biologically different [76]. Although such intratumoral heterogeneity is critical to understanding the disease, traditional cancer cell line models have difficulties recapitulating the complexity [22, 23]. In this study, we established a novel suite of organ-derived metastatic cell lines and subsequently performed a comprehensive transcriptome analysis of cancer progression in relation to CD44 levels. Key pathways relevant to metastatic disease were found to be upregulated in the various cell types, and the main takeaways are diagrammed in Fig. 7.

CD44 expression has been extensively shown to impact breast cancer progression, controlling cellular biology and correlating with certain clinical outcomes [4–6, 17]. However, the exact roles of CD44 remain unclear in breast cancer as both high and low levels of the marker are correlated with tumor-promoting and tumor-suppressing outcomes [19–22]. In this study, we used metastatic organ-derived cell lines to investigate the effects of CD44 expression on cancer progression across different metastatic niches. As previously shown in 4T1 cell lines [77], models of metastatic breast cancer are invaluable for the advancement of the field. The MMTV-PyMT mouse model of breast cancer is the most popular transgenic preclinical system to study mammary tumor progression and metastatic disease translatable to patients [78, 79]. Here, we successfully isolated primary cell lines from four different organ-derived tissues. The cell lines were isolated based on their expression of CD44^low^/EpCAM^high^ or CD44^high^/EpCAM^high^ to examine the role of CD44 in breast cancer across the various metastatic niches (Fig. 7). The cells were expanded to use as a reproducible resource for experiments in this study and beyond. It is possible, though, that the culturing conditions altered some genomic pathways and/or skewed the levels of CD44^low^ or CD44^high^ cells in the samples.

The suite of MMTV-PyMT-derived cell lines was subjected to comprehensive transcriptome analysis using bulk RNA-seq and scRNA-seq. Although there were shared GO terms across all cell lines belonging to either CD44 signature, we identified numerous changes in gene expression that were organ-specific and could promote metastatic homing [52]. Changes in gene expression related to EMT and the metastatic cascade were also identified. As expected, distal metastatic-derived tissues such as the lung and BM had more differentially expressed genes than lymph node-derived cell lines compared to the primary tumor.

Using scRNA-seq on select organ-derived metastatic cells, we found that the cell lines exhibited a range of intratumoral heterogeneity. Although most MMTV-PyMT publications have focused on metastasis from the primary tumor to the lungs, we identified additional distal metastatic cells in the BM. The BM-derived cell lines showed an increase in previously published markers such as *RANKL*, *OPN* (*SPP1*), and *IL2* [4]. Further studies are necessary to better characterize these cells and increase the number of validated BM-derived DEGs (Additional file 1: Fig. 8C). Specifically, it would be prudent to examine differential gene expression within this model compared to other well-established bone-marrow metastatic models of tumor latency and cancer dormancy [4]. Collectively, these transcriptome changes could contribute to sub-clonal evolution during cancer progression across the different metastatic niches.

Although CD44 has traditionally been used to monitor cancer metastasis, recent studies from Gao and coworkers showed that its expression fluctuates during metastatic progression [55]. We observed similar changes in CD44 expression from initial isolates compared to cells used in subsequent transcriptome analysis. Furthermore, recent studies with MDA-MB-231 breast cancer models showed that CD44^low^ cells possessed stem cell like properties that CD44^high^ cells lacked [13, 59]. CD44^low^ populations could regenerate both more CD44^low^ and CD44^high^ cell clusters, whereas CD44^high^ cells could only replicate themselves. We observed a similar increase in stem cell markers in CD44^low^ but not CD44^high^ cells. These results suggest that more research is needed to better understand and characterize CD44 expression in breast cancer.

Although CD44 has been correlated to metastatic progression and CSCs, the fluctuating expression levels of this marker complicate its use as a sole classifier of cellular phenotypes. There are thus many combinations of markers currently used to isolate CSCs, with no one set being universally accepted [13, 57–59]. We examined a handful of CSC markers commonly associated with breast cancer (*ALDH1A1, ABCG2, PGP, FUT4*). CSC-associated markers were found in both the bulk RNA-seq and scRNA-seq data. The expression of the CSC-associated markers was highest in lung-derived CD44^low^ cells compared to CD44^high^ cells. Upregulation of other CSC-associated markers, including the stem cell survival signaling member WNT, was also observed in the CD44low cells, corroborating our hypothesis that these cells possess stem-like properties [17]. scRNA-seq further revealed three distinct clusters for lung-derived CD44^low^ cells on the UMAP. Unlike the lung-derived CD44^high^ groups, lungL1 and lungL2 cells retained properties of stem cells but dramatically increased their expression of mesenchymal-like signaling pathways involved in cancer cell maintenance and dormancy (*TSP, TNC, BMP*) [6, 14, 16]. Interestingly, lungL3 was a much smaller, separate cluster that lacked the dormancy markers and instead expressed high levels of stem cell markers. The diversity of CD44^low^ clusters could be explained by heterogeneity within the different stages of metastatic progression and along the EMT spectrum. Another possibility is that lungL1 and lungL2 clusters comprise cancer-associated fibroblasts, while the lungL3 might be a distinct cluster of CSCs.

Breast cancer progression relies on CSCs, to initiate tumor cell growth during metastatic dissemination and colonization of new organs. Previous work from Weinberg has shown that tumor-initiating cells (TICs) originating in the luminal cell layer of the mammary gland rely on EMT-initiating transcription factors (TFs) for cellular dedifferentiation [56]. These factors activate signaling pathways distinct from TICs originating from the basal mammary gland. Bulk RNA-seq data allowed us to further characterize the lung-derived cell lines based on expression of EMT-TFs known to induce TICs. For example, increased expression of the EMT-TF Snail was observed in lung CD44^low^ cells compared to CD44^high^ cells. Although both CD44 signatures of lung-derived cells expressed the EMT-TF binding promoter and TIC master regulator *ZEB1*, lung-derived CD44^low^ cells also showed a greater increase in the basal cell-associated EMT-TF Slug as compared to CD44^high^ cells. Weinberg and others have shown that Slug and Snail both bind and regulate *ZEB1* expression [56]; however, the expression of *SLUG* is associated with breast cancers that arise from cells containing normal mammary epithelial stem cells in the basal compartment. *SLUG* expression is also associated with highly dedifferentiated breast cancer cells found in the advanced and final stages of metastatic disease. Here, we harvested MMTV-PyMT mice well after the time period sampled by Weinberg. The most striking result from the lung-derived cells was that the stem cell-associated features of tumor-initiating cells were observed most in CD44^low^ opposed to CD44^high^ expressing cells that have previously been correlated to CSCs.

Further work examining the effects of the microenvironment as a key regulator of cellular plasticity adds to the theory that “dedifferentiated” non-CSCs can undergo processes that endow them with CSC-like properties [13, 37]. These complex studies highlight the need for further investigation into the possible origins of CSCs in relation to the surrounding microenvironment. Modification of cellular metabolism along with remodeling of the tumor microenvironment can be achieved through the collective change of different groups of genes. The modulation of certain genes has been shown to subsequently facilitate CSC phenotypes that lead to metastatic colonization. Based on the gene expression changes dictating cellular metabolism and matrix remodeling, we hypothesized that lung-derived CD44^low^ cells could harbor some CSC properties.

To better understand the origins of the CD44 signatures, we examined the scRNA-seq results as they projected over pseudo-time using Monocole2. From the three different cell lines, we identified that lymph node-derived CD44^high^ cells were projected to give rise to lung-derived cells of both signatures. Based on the metastatic cascade of organ tropism, the proximal location of the lymph nodes to the primary tumor is well documented to be the initial site of metastatic lesions before advancing to the more distal lungs. Future studies would benefit from expanding the analysis to include lymph node-derived CD44^low^ cell lines to determine how this signature affects the projection of the tumor-initiating cell population.

## Conclusions

Collectively, we established organ-derived cancer cell lines from different metastatic niches. Comprehensive transcriptomic analysis was performed and revealed the impacts of heterogeneity on cancer progression. Bulk sequencing analyses uncovered tissue-specific genes across the different metastatic and primary tumor samples. We further investigated intratumoral heterogeneity by performing single-cell RNA-seq. These data will improve our understanding of the metastatic cascade and tumor heterogeneity in breast cancer, and potentially reveal new therapeutic targets.

## Supplementary Information

**Additional file 1.** Supplemental figures mentioned in main text.

**Additional file 2**. Complete list of enriched GO-terms and genes from the enriched pathway analysis from Fig 1B.

**Additional file 3**. Differential gene analysis comparing metastatic tissue-derived cell line (CD44^high^ BM) against the MFP-derived cell line (primary tumor).

**Additional file 4**. Differential gene analysis comparing metastatic tissue-derived cell line (CD44^low^ BM) against the MFP-derived cell line (primary tumor).

**Additional file 5**. Differential gene analysis comparing metastatic tissue-derived cell line (CD44^high^ lung) against the MFP-derived cell line (primary tumor).

**Additional file 6**. Differential gene analysis comparing metastatic tissue-derived cell line (CD44^low^ lung) against the MFP-derived cell line (primary tumor).

**Additional file 7**. Differential gene analysis comparing metastatic tissue-derived cell line (CD44^high^ LN) against the MFP-derived cell line (primary tumor).

**Additional file 8**. Differential gene analysis comparing metastatic tissue-derived cell line (CD44^low^ LN) against the MFP-derived cell line (primary tumor)

**Additional file 9**. Differentially expressed gene analysis comparing CD44^low^ and CD44^high^ samples derived from bone marrow.

**Additional file 10**. Differentially expressed gene analysis comparing CD44^low^ and CD44^high^ samples derived from lung tissue.

**Additional file 11**. Differentially expressed gene analysis comparing CD44^low^ and CD44^high^ samples derived from lymph node tissue.

**Additional file 12**. Complete list of enriched GO-terms and upregulated genes for CD44^high^ samples across all tissues (from Fig 3A).

**Additional file 13**. Complete list of enriched GO-terms and upregulated genes for CD44^low^ samples across all tissues (from Fig 3A).

**Additional file 14**. Complete list of enriched GO-terms and upregulated genes from CD44^high^ vs. CD44^low^ lymph node-derived samples (Fig 3B).

**Additional file 15**. Complete list of enriched GO-terms and upregulated genes from CD44^low^ vs. CD44^high^ lymph node-derived samples (Fig 3B).

**Additional file 16**. Complete list of enriched GO-terms and upregulated genes from CD44^high^ vs. CD44^low^ lung-derived samples (Fig 3C).

**Additional file 17**. Complete list of enriched GO-terms and upregulated genes from CD44^low^ vs. CD44^high^ lung-derived samples (Fig 3C).

## Data Availability

Not applicable**.**

## Abbreviations

BM: Bone marrow
CSC: Cancer stem cells
DEGs: Differentially expressed genes
EMT: Epithelial to mesenchymal transition
L: Lungs
LN: Lymph nodes
LungH: Lung-derived CD44^high^
LungH1: Lung-derived CD44^high^ cluster 1
LungH2: Lung-derived CD44^high^ cluster 2
LungH3: Lung-derived CD44^high^ cluster 3
LungL: Lung-derived CD44^low^
LungL1: Lung-derived CD44^low^ cluster 1
LungL2: Lung-derived CD44^low^ cluster 2
LungL3: Lung-derived CD44^low^ cluster 3
LymphH: LN-derived CD44^high^
LymphH1: LN-derived CD44^high^ clusters 1
LymphH2: LN-derived CD44^high^ clusters 2
LymphH3: LN-derived CD44^high^ clusters 3
LymphH4: LN-derived CD44^high^ clusters 4
LymphH5: LN-derived CD44^high^ clusters 5
LymphH6: LN-derived CD44^high^ clusters 6
LymphH7: LN-derived CD44^high^ clusters 7
MET: Mesenchymal to epithelial transition
MFP: Mammary fat pad
RA: Retinoic acid
RNA-seq: Bulk RNA-sequencing
scRNA-seq: Single-cell RNA-seq
TFs: Transcription factors
TICs: Tumor-initiating cells

## References

[1] Waks AG, Winer EP (2019). Breast cancer treatment: a review. JAMA.

[2] Vanharanta S, Massague J (2013). Origins of metastatic traits. Cancer Cell.

[3] Steeg PS (2016). Targeting metastasis. Nat Rev Cancer.

[4] Hanahan D, Weinberg RA (2011). Hallmarks of cancer: the next generation. Cell.

[5] Harbeck N (2019). Breast cancer. Nat Rev Dis Primers.

[6] Montagner M, Sahai E (2020). In vitro models of breast cancer metastatic dormancy. Front Cell Dev Biol.

[7] Cai Y (2017). Transcriptomic dynamics of breast cancer progression in the MMTV-PyMT mouse model. BMC Genom.

[8] Zheng W (2019). Lung mammary metastases but not primary tumors induce accumulation of atypical large platelets and their chemokine expression. Cell Rep.

[9] Olsen TK, Baryawno N (2018). Introduction to single-cell RNA sequencing. Curr Protoc Mol Biol.

[10] Yeo S.K. et al. Single-cell RNA-sequencing reveals distinct patterns of cell state heterogeneity in mouse models of breast cancer. Elife 2020;9. 10.7554/eLife.58810 PMC7447441 32840210

[11] Li H (2017). Reference component analysis of single-cell transcriptomes elucidates cellular heterogeneity in human colorectal tumors. Nat Genet.

[12] Perlman RL (2016). Mouse models of human disease: an evolutionary perspective. Evol Med Public Health.

[13] Li Y, Laterra J (2012). Cancer stem cells: Distinct entities or dynamically regulated phenotypes?. Cancer Res.

[14] De Angelis ML (2019). Stem cell plasticity and dormancy in the development of cancer therapy resistance. Front Oncol.

[15] Takebe N, Ivy SP (2010). Controversies in cancer stem cells: targeting embryonic signaling pathways. Clin Cancer Res.

[16] Ghajar CM (2013). The perivascular niche regulates breast tumour dormancy. Nat Cell Biol.

[17] Ma J (2012). Characterization of mammary cancer stem cells in the MMTV-PyMT mouse model. Tumour Biol.

[18] Kersten K, Salvagno C, de Visser KE (2015). Exploiting the immunomodulatory properties of chemotherapeutic drugs to improve the success of cancer immunotherapy. Front Immunol.

[19] Crabtree JS, Miele L. Breast cancer stem cells. Biomedicines. 2018;6(3):94. 10.3390/biomedicines6030077 PMC6163894 30018256

[20] Fico F (2019). Breast cancer stem cells with tumor- versus metastasis-initiating capacities are modulated by TGFBR1 inhibition. Stem Cell Rep.

[21] Lobba AR (2012). Differential expression of CD90 and CD14 stem cell markers in malignant breast cancer cell lines. Cytometry A.

[22] Haynes B (2017). Breast cancer complexity: implications of intratumoral heterogeneity in clinical management. Cancer Metastasis Rev.

[23] Liu J, Dang H, Wang XW (2018). The significance of intertumor and intratumor heterogeneity in liver cancer. Exp Mol Med.

[24] Gomez-Cuadrado L (2017). Mouse models of metastasis: progress and prospects. Dis Model Mech.

[25] Lawson DA (2015). Single-cell analysis reveals a stem-cell program in human metastatic breast cancer cells. Nature.

[26] Liao X, Makris M, Luo XM (2016). Fluorescence-activated cell sorting for purification of plasmacytoid dendritic cells from the mouse bone marrow. J Vis Exp..

[27] Marhaba R (2008). CD44 and EpCAM: cancer-initiating cell markers. Curr Mol Med.

[28] Guy CT, Cardiff RD, Muller WJ (1992). Induction of mammary tumors by expression of polyomavirus middle T oncogene: a transgenic mouse model for metastatic disease. Mol Cell Biol.

[29] Dobin A (2013). STAR: ultrafast universal RNA-seq aligner. Bioinformatics.

[30] Li B, Dewey CN (2011). RSEM: accurate transcript quantification from RNA-Seq data with or without a reference genome. BMC Bioinf.

[31] Robinson MD, McCarthy DJ, Smyth GK (2010). edgeR: a Bioconductor package for differential expression analysis of digital gene expression data. Bioinformatics.

[32] Bray NL (2016). Near-optimal probabilistic RNA-seq quantification. Nat Biotechnol.

[33] Hen O, Barkan D (2020). Dormant disseminated tumor cells and cancer stem/progenitor-like cells: Similarities and opportunities. Semin Cancer Biol.

[34] Cardoso F (2016). 70-gene signature as an aid to treatment decisions in early-stage breast cancer. N Engl J Med.

[35] van 't Veer LJ, et al. Gene expression profiling predicts clinical outcome of breast cancer. Nature. 2002;415(6871):530–6. 10.1038/415530a 11823860

[36] Institute NC. Diagnosis and staging. About Cancer 2019 [cited 2020; Available from: https://www.cancer.gov/about-cancer/diagnosis-staging/diagnosis.

[37] Takebe N, Warren RQ, Ivy SP (2011). Breast cancer growth and metastasis: interplay between cancer stem cells, embryonic signaling pathways and epithelial-to-mesenchymal transition. Breast Cancer Res.

[38] Feng Y (2018). Breast cancer development and progression: risk factors, cancer stem cells, signaling pathways, genomics, and molecular pathogenesis. Genes Dis.

[39] Evans MK (2016). X-linked inhibitor of apoptosis protein mediates tumor cell resistance to antibody-dependent cellular cytotoxicity. Cell Death Dis.

[40] Altieri DC (2008). Survivin, cancer networks and pathway-directed drug discovery. Nat Rev Cancer.

[41] Wasinski B (2020). Discoidin domain receptors, DDR1b and DDR2, promote tumour growth within collagen but DDR1b suppresses experimental lung metastasis in HT1080 xenografts. Sci Rep.

[42] Xu X (2005). Matrix metalloproteinase-2 contributes to cancer cell migration on collagen. Cancer Res.

[43] Morandi EM (2016). ITGAV and ITGA5 diversely regulate proliferation and adipogenic differentiation of human adipose derived stem cells. Sci Rep.

[44] Heldin CH (2013). Targeting the PDGF signaling pathway in tumor treatment. Cell Commun Signal.

[45] Atsumi T (2002). High expression of inducible 6-phosphofructo-2-kinase/fructose-2,6-bisphosphatase (iPFK-2; PFKFB3) in human cancers. Cancer Res.

[46] Li N, Spetz MR, Ho M (2020). The role of glypicans in cancer progression and therapy. J Histochem Cytochem.

[47] Nguyen DX, Bos PD, Massague J (2009). Metastasis: from dissemination to organ-specific colonization. Nat Rev Cancer.

[48] Minn AJ (2005). Genes that mediate breast cancer metastasis to lung. Nature.

[49] Obenauf AC, Massague J (2015). Surviving at a distance: organ-specific metastasis. Trends Cancer.

[50] Westbrook JA (2016). CAPG and GIPC1: breast cancer biomarkers for bone metastasis development and Treatment. J Natl Cancer Inst..

[51] Sun J (2019). Overexpression of CENPF correlates with poor prognosis and tumor bone metastasis in breast cancer. Cancer Cell Int.

[52] Chen W (2018). Organotropism: new insights into molecular mechanisms of breast cancer metastasis. NPJ Precis Oncol.

[53] Ross C (2020). Metastasis-specific gene expression in autochthonous and allograft mouse mammary tumor models: stratification and identification of targetable signatures. Mol Cancer Res.

[54] Wood SL, Brown JE (2020). Personal medicine and bone metastases: biomarkers, micro-RNAs and bone metastases. Cancers (Basel)..

[55] Yang C (2019). Inducible formation of leader cells driven by CD44 switching gives rise to collective invasion and metastases in luminal breast carcinomas. Oncogene.

[56] Ye X (2015). Distinct EMT programs control normal mammary stem cells and tumour-initiating cells. Nature.

[57] Nowell PC (1976). The clonal evolution of tumor cell populations. Science.

[58] Sachidanandam R, et al. A map of human genome sequence variation containing 1.42 million single nucleotide polymorphisms. Nature 2001;409(6822):928–33. 10.1038/35057149 11237013

[59] Chen K, Fraley SI (2020). Abstract 5706: Identifying regulators of cancer heterogeneity with phenotypic sorting and single cell sequencing. Can Res..

[60] Weigelt B, Peterse JL, van 't Veer LJ. Breast cancer metastasis: markers and models. Nat Rev Cancer. 2005;5(8):591–602. 10.1038/nrc1670 16056258

[61] Quail DF, Joyce JA (2013). Microenvironmental regulation of tumor progression and metastasis. Nat Med.

[62] Nagai T (2020). Tactics of cancer invasion: solitary and collective invasion. J Biochem.

[63] Huang T (2017). Thrombospondin-1 is a multifaceted player in tumor progression. Oncotarget.

[64] Iliopoulos D (2011). Inducible formation of breast cancer stem cells and their dynamic equilibrium with non-stem cancer cells via IL6 secretion. Proc Natl Acad Sci USA.

[65] Plaks V, Kong N, Werb Z (2015). The cancer stem cell niche: HOW essential is the niche in regulating stemness of tumor cells?. Cell Stem Cell.

[66] Yi Y (2020). Transcriptional suppression of AMPKalpha1 promotes breast cancer metastasis upon oncogene activation. Proc Natl Acad Sci USA.

[67] Yang L (2020). Targeting cancer stem cell pathways for cancer therapy. Signal Transduct Target Ther.

[68] Tomita H (2016). Aldehyde dehydrogenase 1A1 in stem cells and cancer. Oncotarget.

[69] Vassalli G (2019). Aldehyde dehydrogenases: not just markers, but functional regulators of stem cells. Stem Cells Int.

[70] Barney LE (2020). Tumor cell-organized fibronectin maintenance of a dormant breast cancer population. Sci Adv..

[71] Kim RS, et al. Dormancy signatures and metastasis in estrogen receptor positive and negative breast cancer. PLoS ONE 2012;7(4):e35569. 10.1371/journal.pone.0035569 PMC3329481 22530051

[72] Sosa MS, Bragado P, Aguirre-Ghiso JA (2014). Mechanisms of disseminated cancer cell dormancy: an awakening field. Nat Rev Cancer.

[73] Ibrahim SA (2017). Syndecan-1 is a novel molecular marker for triple negative inflammatory breast cancer and modulates the cancer stem cell phenotype via the IL-6/STAT3, Notch and EGFR signaling pathways. Mol Cancer.

[74] Gotte M (2006). Predictive value of syndecan-1 expression for the response to neoadjuvant chemotherapy of primary breast cancer. Anticancer Res.

[75] Grimm D (2020). The role of SOX family members in solid tumours and metastasis. Semin Cancer Biol.

[76] Martelotto LG (2014). Breast cancer intra-tumor heterogeneity. Breast Cancer Res.

[77] Aslakson CJ, Miller FR (1992). Selective events in the metastatic process defined by analysis of the sequential dissemination of subpopulations of a mouse mammary tumor. Cancer Res.

[78] Lifsted T (1998). Identification of inbred mouse strains harboring genetic modifiers of mammary tumor age of onset and metastatic progression. Int J Cancer.

[79] Le Voyer T (2000). An epistatic interaction controls the latency of a transgene-induced mammary tumor. Mamm Genome.

