# Supplementary material for: Transcriptome analysis of heterogeneity in mouse model of metastatic breast cancer
Source: Breast Cancer Res. 2021 Sep 27;23:93. doi: 10.1186/s13058-021-01468-x (PMC8477508; doi:10.1186/s13058-021-01468-x)
Supplement: Supplementary file 1 — Additional file 1. Supplemental figures mentioned in main text. [file 13058_2021_1468_MOESM1_ESM.pdf]

Supporting Information

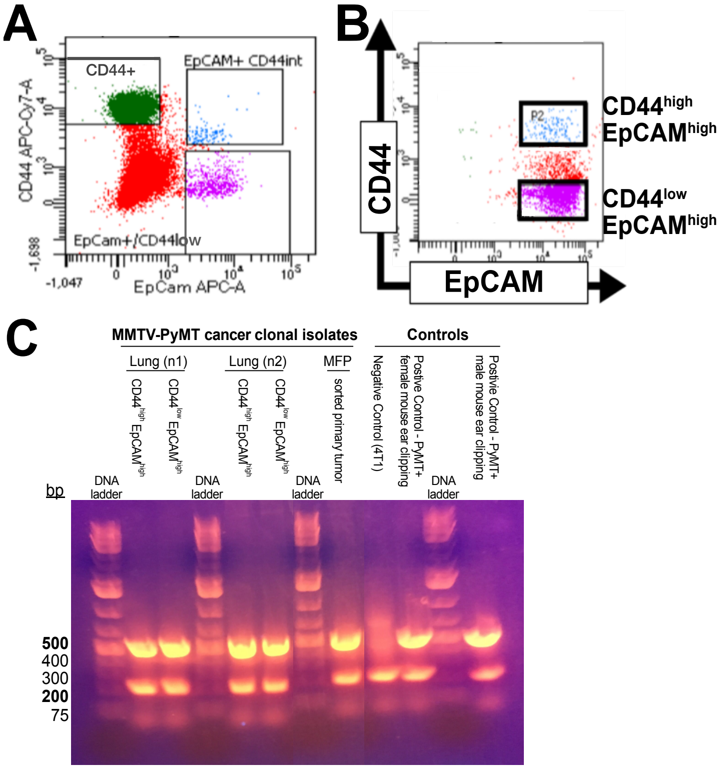

SI Figure 1. Metastatic cell line derivation and validation. (A) Representative flow cytometry plot for metastatic tissue-derived isolates prior to sorting. The desired CD44^{low}/EpCAM^{high} and CD44^{high}/EpCAM^{high} cell populations and associated gates are highlighted. These cells were sorted and for transcriptomic analysis. **(B)** Clonal isolates from **(A)** recapitulate metastatic disease in vivo. Sorted cells were reimplanted into disease-free FVB mice. Once tumors formed, distal organs were collected and examined for metastatic lesions. A representative FACS plot for metastatic cells harvested from lymph node tissue upon tumor reimplantation in FVB mice. The expected CD44^{low}/EpCAM^{high} and CD44^{high}/EpCAM^{high} populations are highlighted. **(C)** PCR was used to confirm the presence of PyMT viral antigen using gDNA extract from cell lines and controls. Samples with amplified 500bp and 200bp bands are positive for PyMT antigen expression.

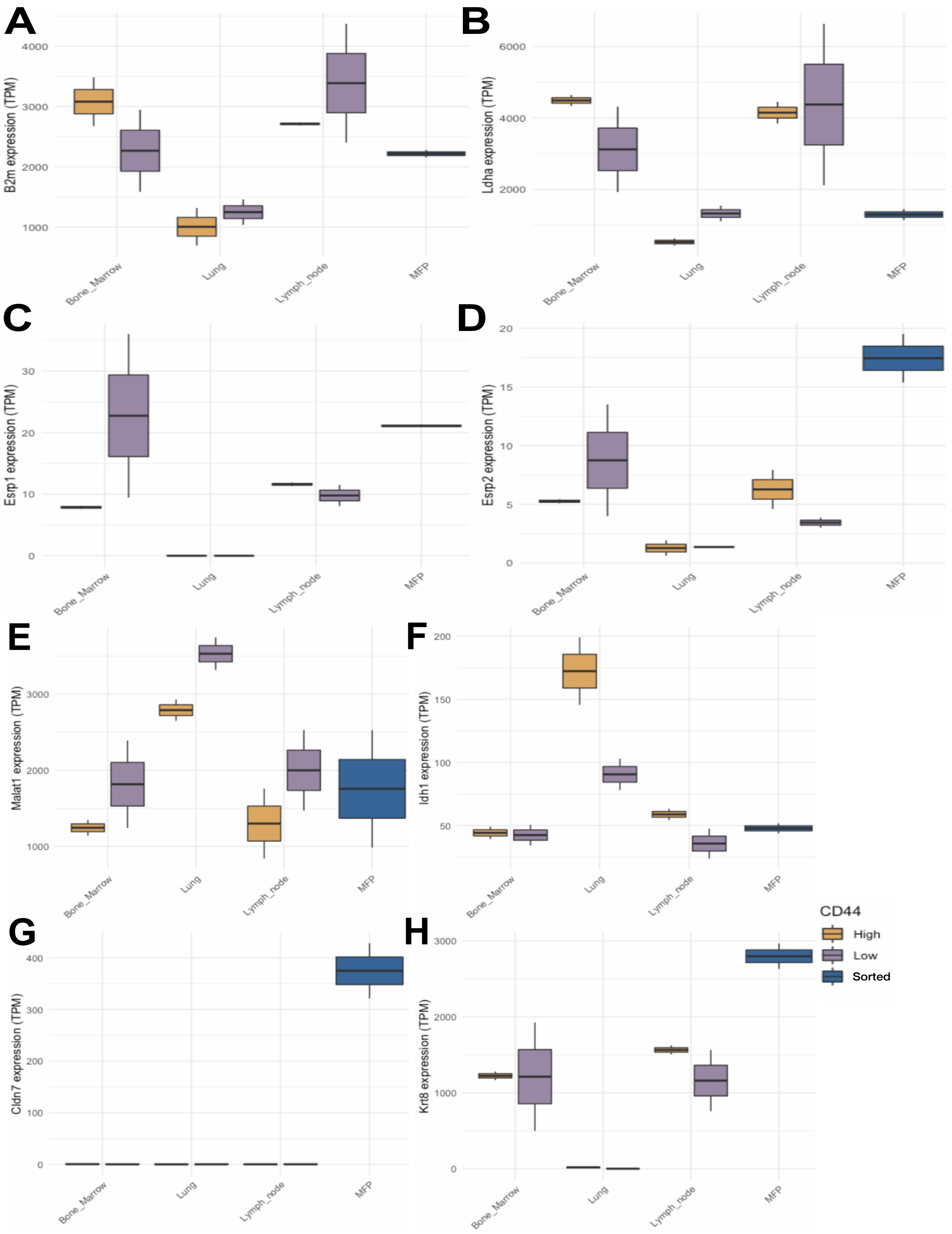

SI Figure 2. Established breast cancer transcripts identified in MMTV-PyMT cell lines. Bulk RNA sequencing analysis of bone marrow, lung, and lymph node-derived cell lines was performed and transcript levels for a panel of breast cancer markers were measured. For (A-H), expression of gene transcripts (TPM, transcript per million) for CD44 high expressing cells (yellow), CD44 low expressing cells (purple), and MFP CD44 expressing cells (blue) are shown.

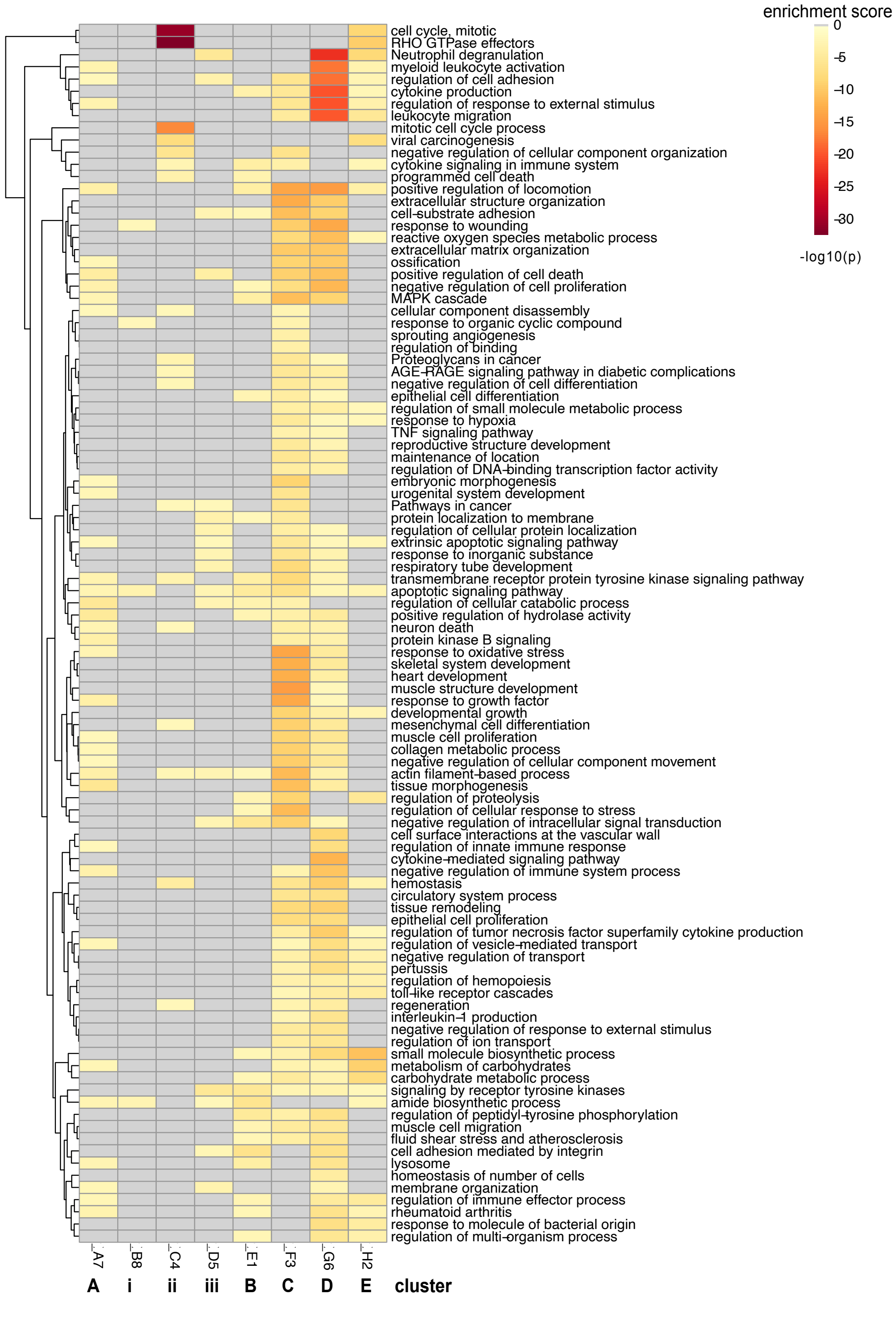

SI Figure 3. Complete list of enriched GO-terms and pathway analysis from Fig 1D. Heat map shows the enrichment scores of the pathways for each cluster (A-E; i-iii).

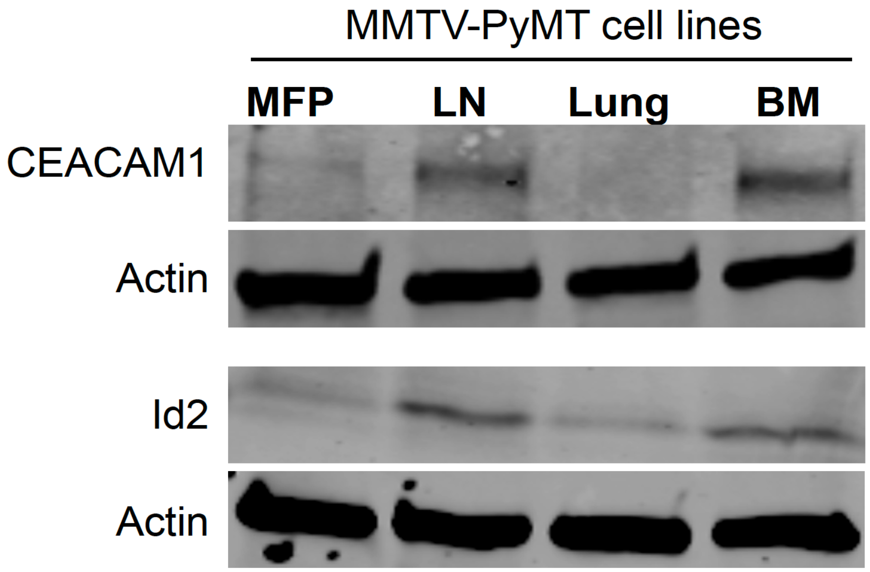

SI Figure 4. Western blot analysis of markers identified from RNA-seq analysis. Select markers were selected from among the DEGs shown in Fig 1D. The following proteins were examined: Id2 (predicted to be highly expressed in lymph node-derived cell lines) and CEACAM1 (predicted to be highly expressed in bone marrow-derived cell lines). Actin was used as a loading control.

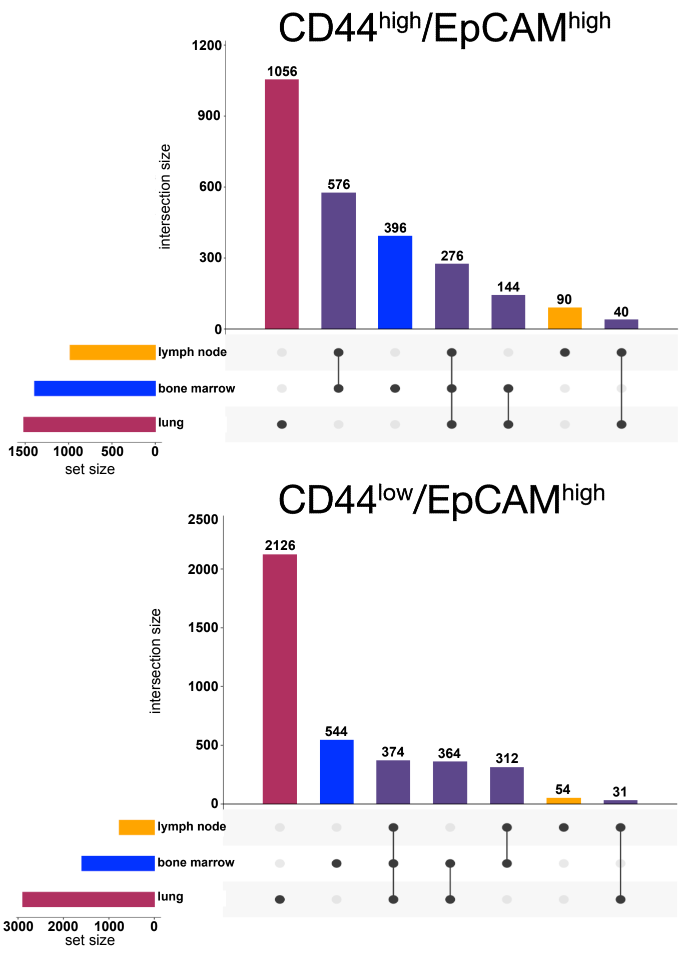

SI Figure 5. Box and whisker plot comparing the number of upregulated genes in CD44^{high}/EpCAM^{high} (top plot) and CD44^{low}/EpCAM^{high} (bottom plot) expressing cells from the different metastatic tissues of origin. Distinct gene expression signatures are shown with single black dots corresponding to a specific tissue-derived clonal isolate. Genes that are shared between different metastatic sites are represented by black lines that connect the specific samples. The total number of upregulated genes are denoted above each bar on the graph.

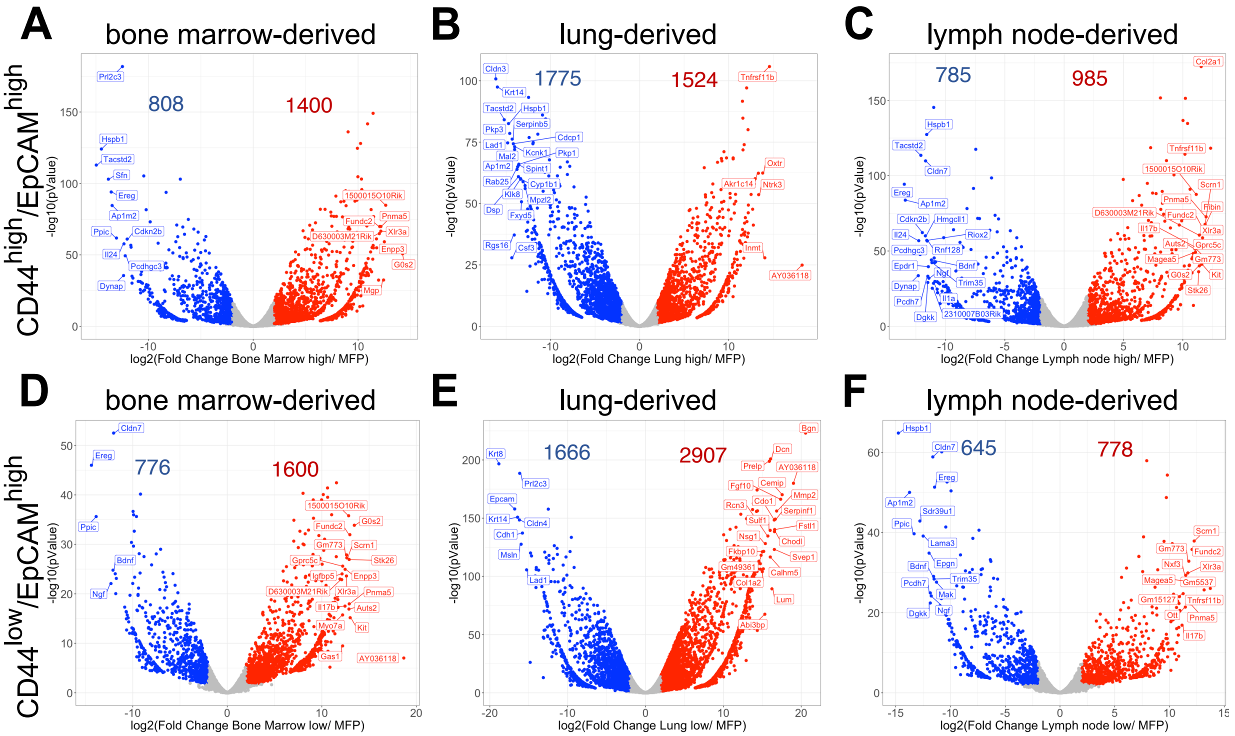

SI Figure 6. Volcano plots of DEGs from tissue-derived metastatic cell lines. (A-C) Volcano plots of DEGs from tissue-derived metastatic cell lines with CD44^{high}/EpCAM^{high} expression compared to primary tumor samples. **(A)** Upregulated genes in bone marrow-derived (BM) isolates with CD44^{high}/EpCAM^{high} expression are shown in red (1,400 genes), while 808 genes (blue) were upregulated in the primary tumor. **(B)** Upregulated genes in lung-derived isolates with CD44^{high}/EpCAM^{high} expression are shown in red (1,524 genes), while 1,775 genes (blue) were upregulated in the primary tumor. **(C)** Upregulated genes in lymph node-derived (LN) isolates with CD44^{high}/EpCAM^{high} expression are shown in red (985 genes), while 785 genes (blue) were upregulated in the primary tumor. **(D-F)** Volcano plots of differentially expressed genes from tissue-derived metastatic cell lines with CD44^{low}/EpCAM^{high} expression when compared to primary tumor samples. **(D)** Upregulated genes in bone marrow-derived (BM) isolates with CD44^{low}/EpCAM^{high} expression are shown in red (1,600 genes), while 776 genes (blue) were upregulated in the primary tumor. **(E)** Upregulated genes in lung-derived isolates with CD44^{low}/EpCAM^{high} expression are shown in red (2,907 genes), while 1666 genes (blue) were upregulated in the primary tumor. **(F)** Upregulated genes in lymph node-derived (LN) isolates with CD44^{low}/EpCAM^{high} expression are shown in red (778 genes), while 645 genes (blue) were upregulated in the primary tumor. The fewer differentially expressed genes amongst the tissue-derived isolates compared to the primary tumor is suggestive of the order in the metastatic cascade with LN-derived isolates bring the first metastatic site.

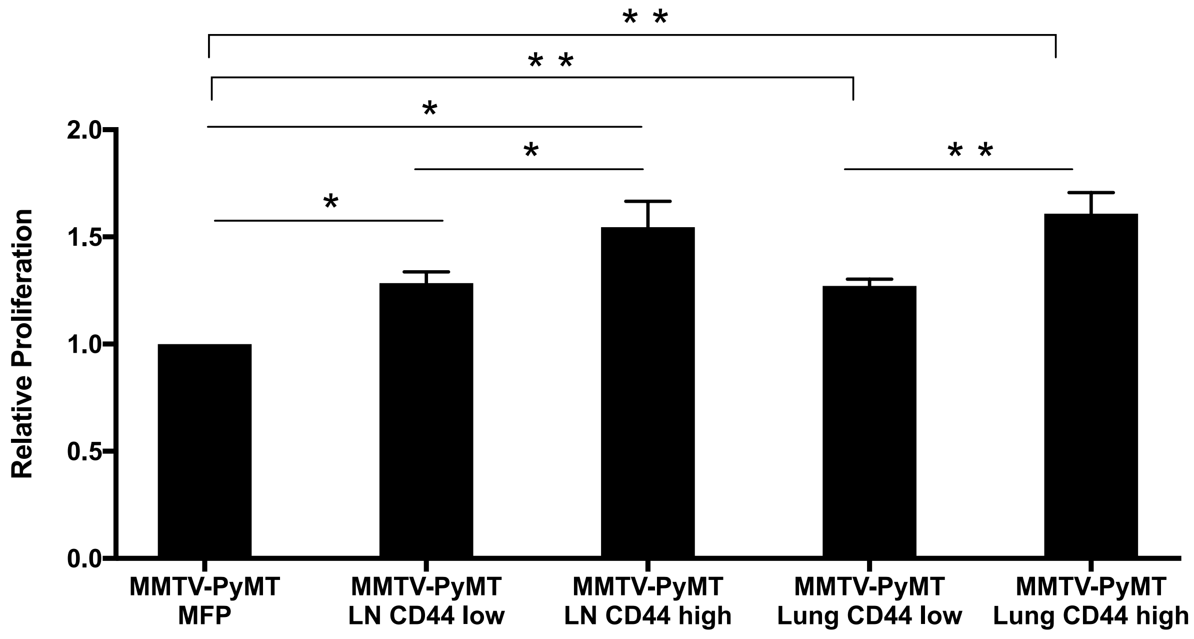

SI Figure 7. Cell proliferation analysis. MMTV-PyMT cell growth was measured over 24 h using a crystal violet assay. Relative proliferation values for lymph node (LN) and lung-derived cells (compared to MFP-derived cells) are shown. Error bars represent the standard deviation of the mean for n=3 replicates. ** p < 0.01; * p < 0.05

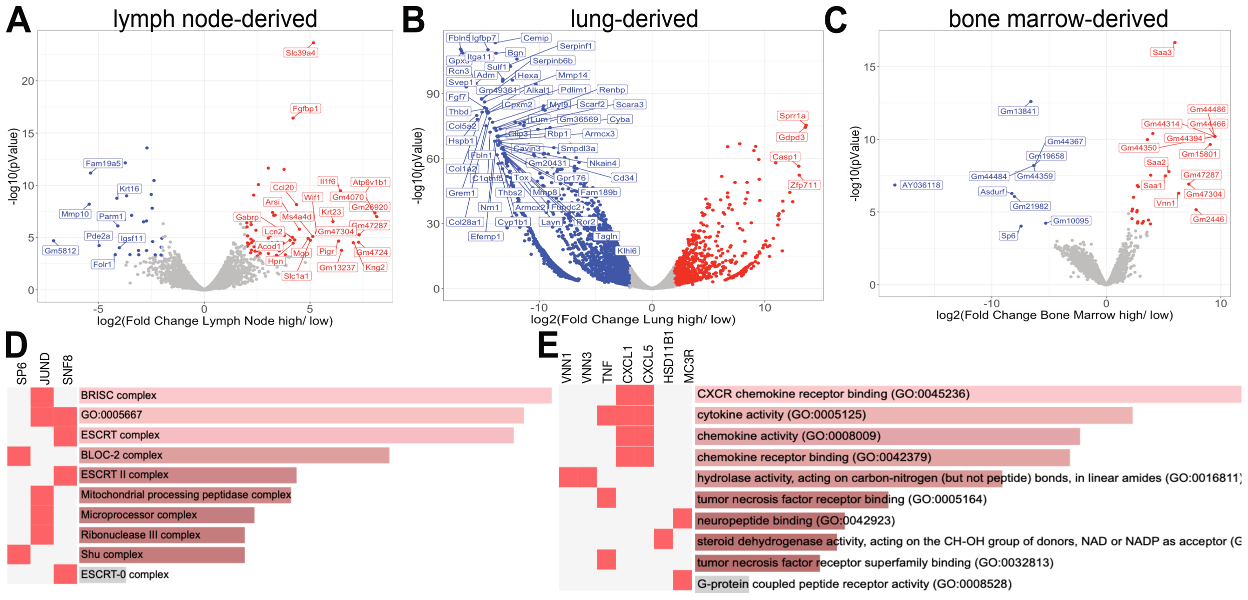

SI Figure 8. Analysis of DEGs from metastatic isolates based on CD44 expression. (A) Volcano plots showing DEGs in CD44^{high}/EpCAM^{high} (red) versus CD44^{low}/EpCAM^{high} (blue) from **(A)** lymph node-derived, **(B)** lung-derived, and **(C)** bone marrow-derived metastatic cells. **(D)** GO terms and relevant genes upregulated in CD44^{high}/EpCAM^{high} bone marrow-derived cells. **(E)** GO terms and relevant genes upregulated in CD44^{low}/EpCAM^{high} bone marrow-derived cells.

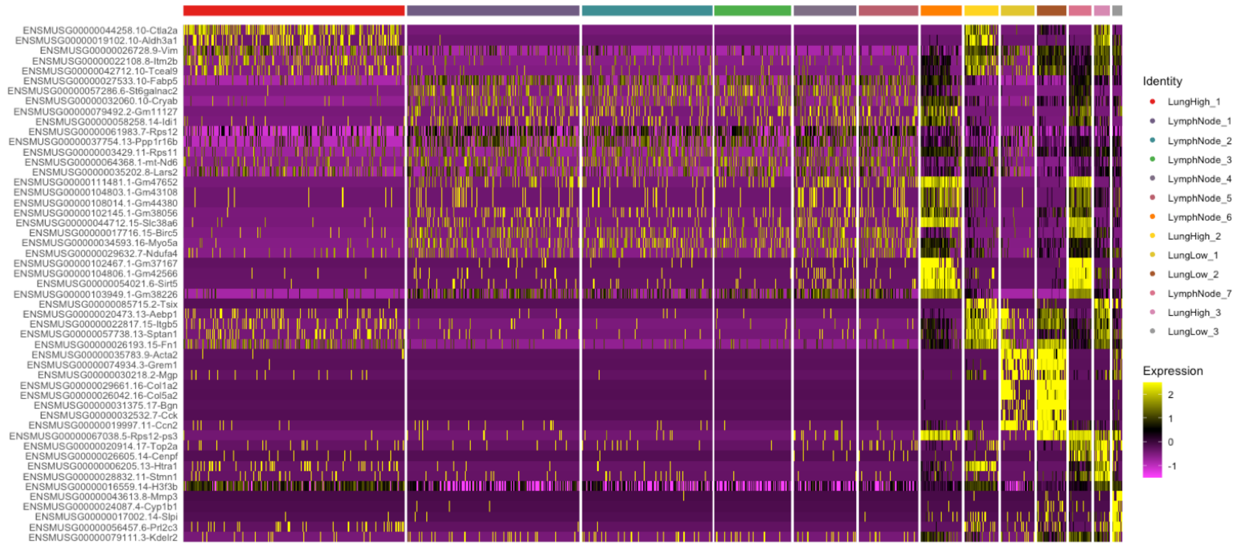

SI Figure 9. Hierarchical clustering analysis from single cell sequencing analyses. Single cell sequencing heat map showing 13 tissue-specific clusters. See Satija, *et al.* for details on the method.

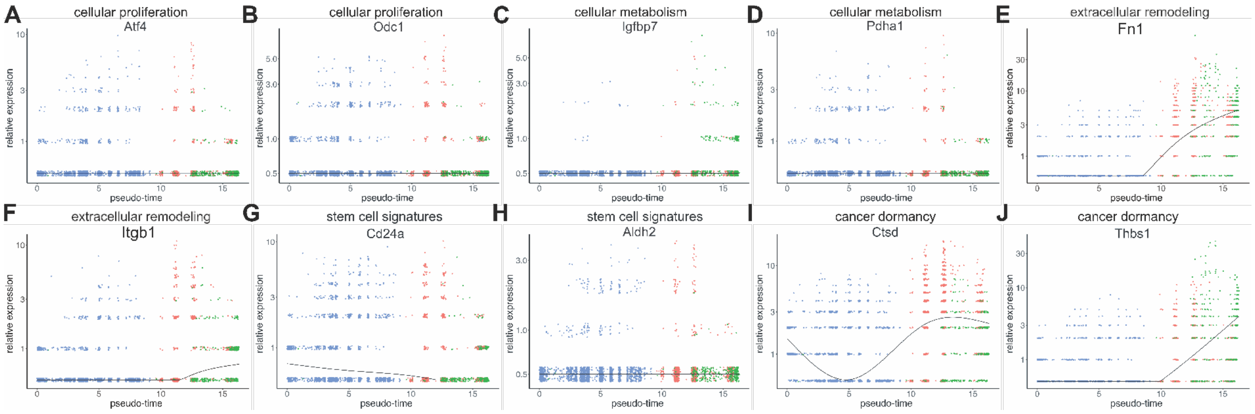

SI Figure 10. Monocle2 pseudo-time analysis of single cells. The metastatic trajectory of distinct cells clusters is shown. The composition of the cells was identified by coloring the pseudo-time map with the tissue of origins (as in Fig. 5A). Expression of cellular proliferation markers **(A, B)**, metabolism markers (**C, D)**, extracellular remodeling markers **(E,F)**, stem cell signatures **(G,H)**, and cancer dormancy markers **(I,J)** of single cells across pseudo-time. Trend line on graphs tracks the statistical significance of gene expression as it changes across pseudo-time. See Qiu, *et al*. for details on Monocle2.

References

Bray, N. L., Pimentel, H., Melsted, P., & Pachter, L. (2016). Near-optimal probabilistic RNA-seq quantification. *Nat Biotechnol, 34*(5), 525-527. doi:10.1038/nbt.3519

Dobin, A., *et al*. (2013). STAR: ultrafast universal RNA-seq aligner. *Bioinformatics, 29*(1), 15-21. doi:10.1093/bioinformatics/bts635

Guy, C. T., Cardiff, R. D., & Muller, W. J. (1992). Induction of mammary tumors by expression of polyomavirus middle T oncogene: a transgenic mouse model for metastatic disease. *Mol Cell Biol, 12*(3), 954-961. doi:10.1128/mcb.12.3.954

Lawson, D. A., *et al*. (2015). Single-cell analysis reveals a stem-cell program in human metastatic breast cancer cells. *Nature, 526*(7571), 131-135. doi:10.1038/nature15260

Li, B., & Dewey, C. N. (2011). RSEM: accurate transcript quantification from RNA-Seq data with or without a reference genome. *BMC Bioinformatics, 12*, 323. doi:10.1186/1471-2105-12-323

Liao, X., Makris, M., & Luo, X. M. (2016). Fluorescence-activated Cell Sorting for Purification of Plasmacytoid Dendritic Cells from the Mouse Bone Marrow. *J Vis Exp*(117). doi:10.3791/54641

Marhaba, R., Klingbeil, P., Nuebel, T., Nazarenko, I., Buechler, M. W., & Zoeller, M. (2008). CD44 and EpCAM: cancer-initiating cell markers. *Curr Mol Med, 8*(8), 784-804. doi:10.2174/156652408786733667

Qiu, X., Mao, Q., Tang, Y. *et al* (2017). Reversed graph embedding resolves complex single-cell trajectories. *Nat Methods* 14, 979–982.

Robinson, M. D., McCarthy, D. J., & Smyth, G. K. (2010). edgeR: a Bioconductor package for differential expression analysis of digital gene expression data. *Bioinformatics, 26*(1), 139-140. doi:10.1093/bioinformatics/btp616

Satija, R., Farrell, J., Gennert, D. *et al* (2015). Spatial reconstruction of single-cell gene expression data. *Nat Biotechnol* 33, 495–502.

Schor, S. L., & Court, J. (1979). Different mechanisms in the attachment of cells to native and denatured collagen. *J Cell Sci, 38*, 267-281.
